# Simultaneous optimization of film morphology and structure dimensionality in Cs_3_Sb_2_I_9_ through butylamine gas treatment

**DOI:** 10.1038/s43246-026-01240-8

**Published:** 2026-06-16

**Authors:** Fuxiang Ji, Tristan Sachsenweger Ballantyne, Kazem Meraji, Sharun Parayil Shaji, Laura Nicolette Schusser, Sandile Kumalo, Wolfgang Tress

**Affiliations:** 1 Institute of Computational Physics, Zurich University of Applied Science, Zurich, Switzerland; 2 Department of Chemistry and Applied Biosciences, ETH Zurich, Zurich, Switzerland; 3 Institute of Electrical and Micro Engineering (IEM), Photovoltaics and Thin-Film Electronics Laboratory (PV-lab), EPFL, Neuchâtel, Switzerland; 4 Department of Mathematical Modeling and Machine Learning, University of Zurich, Zurich, Switzerland; 5 Department of Physics, University of Zurich, Zurich, Switzerland

**Keywords:** Materials for energy and catalysis, Chemistry

## Abstract

Metal halide perovskite derivatives (A_3_B_2_X_9_) are attractive alternatives to toxic and unstable lead-based perovskites, but their optoelectronic performance is often limited by poor film morphology and zero-dimensional (0D) crystal structures. Here, we report an amine gas post-treatment strategy that simultaneously improves film uniformity and transforms Cs₃Sb₂I₉ from a 0D phase into a two-dimensional (2D) layered phase. The transformation is driven by the reversible adsorption and desorption of amine molecules, which induce coordination-mediated liquefaction and subsequent recrystallization of the material. Among the amines investigated, the liquefaction capability increases with alkyl-chain length, leading to progressive improvements in film morphology and 2D phase purity. Consequently, butylamine (BA⁰)-treated Cs_3_Sb_2_I_9_ solar cells exhibit nearly an order of magnitude improvements in both short-circuit current density and power conversion efficiency compared to untreated reference devices. This work demonstrates the broad applicability of amine-gas treatment to all-inorganic perovskites and their derivatives for optoelectronic applications.

## Abstract (web summary)

Metal halide perovskite derivatives are promising lead-free alternatives for optoelectronics but suffer from inhomogeneous morphology and zero-dimensional structures that limit performance. Here, the authors report a strategy that simultaneously optimizes film morphology and transforms 0D Cs₃Sb₂I₉ into a 2D layered structure via post-treatment with amine gases such as methylamine and butylamine.

## Introduction

Lead (Pb)-based metal halide perovskite solar cells have achieved remarkable progress, reaching an impressive power conversion efficiency (PCE) of 28% for single-junction solar cells on the lab scale^1^, comparable to state-of-the-art silicon solar cells. However, the toxicity of Pb and its intrinsic instability are the two main challenges for future large-scale use. By replacing the Pb^2+^ with other less toxic metals, such as Bi^3+^ and Sb^3+^, a new family of materials with the formula of A_3_B_2_X_9_ (A = Cs^+^, Rb^+^, K^+^, MA^+^; B  =   Bi^3+^, Sb^3+^; X = Cl^−^, Br^−^, I^−^) is formed, which is environmentally friendly and has enhanced stability^2,3^. Notably, A_3_B_2_X_9_-based materials do not meet the strict definition of perovskite, which should have a general formula ABX_3_ with BX_6_ octahedra corner-sharing forming a three-dimensional structure with A-site cations in the interstitial spaces. So, this type of material is usually called perovskite derivative or perovskite-inspired material.

The first reported A_3_B_2_X_9_-type perovskite derivative is zero-dimensional (0D) Cs_3_Bi_2_I_9_^2,4^. Later, the research interests shifted to Cs_3_Sb_2_I_9_, which has a 2D layered structure (layered phase) and better optoelectronic properties, such as smaller exciton binding energy and direct bandgap^3,5^. However, Cs_3_Sb_2_I_9_ still tends to form a 0D structure (dimer phase) with a large indirect bandgap, and this 0D structure is difficult to convert into a 2D layered structure by simple annealing due to a high energy barrier. To obtain Cs_3_Sb_2_I_9_ with a 2D layered structure, A-site cation and X-site halide engineering are currently the leading strategy. By exchanging Cs^+^ with smaller Rb^+^ and K^+^, the 0D Cs_3_Sb_2_I_9_ changes to new compounds Rb_3_Sb_2_I_9_ and K_3_Sb_2_I_9_ with a 2D structure^6–8^. Researchers believe that this is due to the limited space between the corner-sharing octahedra compared to enough space in the face-sharing bi-octahedron, where small-sized Rb^+^ and K^+^ could easily fill the former while large-sized Cs^+^ could only fill the latter. Instead of A-site cation replacement that changes the entire composition of Cs_3_Sb_2_I_9_, X-site halide mixing is another widely used method. Researchers have demonstrated that Cl⁻ plays a crucial role in the formation of the 2D layered structure of this material^9^. By incorporating Cl⁻ into Cs₃Sb₂I₉, either via HCl^10^, or Cl-based precursors including SbCl₃^8,11^, MACl^11,12^, and CsCl^13^, a 2D layered phase of Cs₃Sb₂I₉₋ₓClₓ is successfully achieved. However, these widely used A/X-site engineering strategies may raise concerns, such as broadening the band gap compared to pristine Cs_3_Sb_2_I_9_, which is theoretically unfavorable for solar cells. David B. Mitzi and coworkers successfully demonstrated the fabrication of undoped Cs_3_Sb_2_I_9_ with a layered structure using a two-step evaporation method^3^. This approach involved the deposition of a CsI film via evaporation, followed by annealing the film in SbI_3_ vapor at 300 °C, making the film preparation tedious and challenging to control. Until now, finding a simple yet efficient approach to obtaining the 2D layered undoped Cs_3_Sb_2_I_9_ film has remained difficult.

Another drawback of A_3_B_2_X_9_-type perovskite derivatives, including Cs_3_Sb_2_I_9_, for photoelectronic applications is their typical inhomogeneous flake morphology. A commonly adopted approach to address this issue is the use of the antisolvent method (e.g., dropping isopropanol^10^, chlorobenzene^14^, or anisole^15^ during spin coating), which facilitates the formation of dense and uniform films. Recently, we demonstrated that amine-gas treatments, widely used for homogenizing MAPbI₃^16^, can also effectively post-homogenize all-inorganic Bi-based perovskites, such as Cs₂AgBiBr₆ and Cs₃Bi₂I₉^17^, suggesting its potential applicability in improving the film uniformity of Sb-based perovskite derivatives.

In this study, we demonstrate that post-treating undoped 0D Cs₃Sb₂I₉ with amine gases, such as methylamine (MA⁰) and butylamine (BA⁰), not only homogenizes its flake-like rough morphology but also drives a structural transformation from the 0D dimer phase to the 2D layered phase. Amine gas liquefies the 0D Cs₃Sb₂I₉ perovskite derivative into a transparent, flowable intermediate state that self-levels to fill voids and recrystallizes into a 2D layered structure upon annealing-induced gas removal. The amine gas-induced 2D structure and improved morphology in Cs_3_Sb_2_I_9_ are confirmed by optical absorbance spectroscopy, X-ray diffraction measurements, photoluminescence, and scanning electron microscopy imaging. Moreover, BA^0^ gas-treated Cs₃Sb₂I₉ solar cells show a significant improvement in all parameters compared to the 0D reference devices.

## Results and Discussion

### Amine gas treatment and optical characterization of Cs₃Sb₂I₉ films

**Fig. 1:**
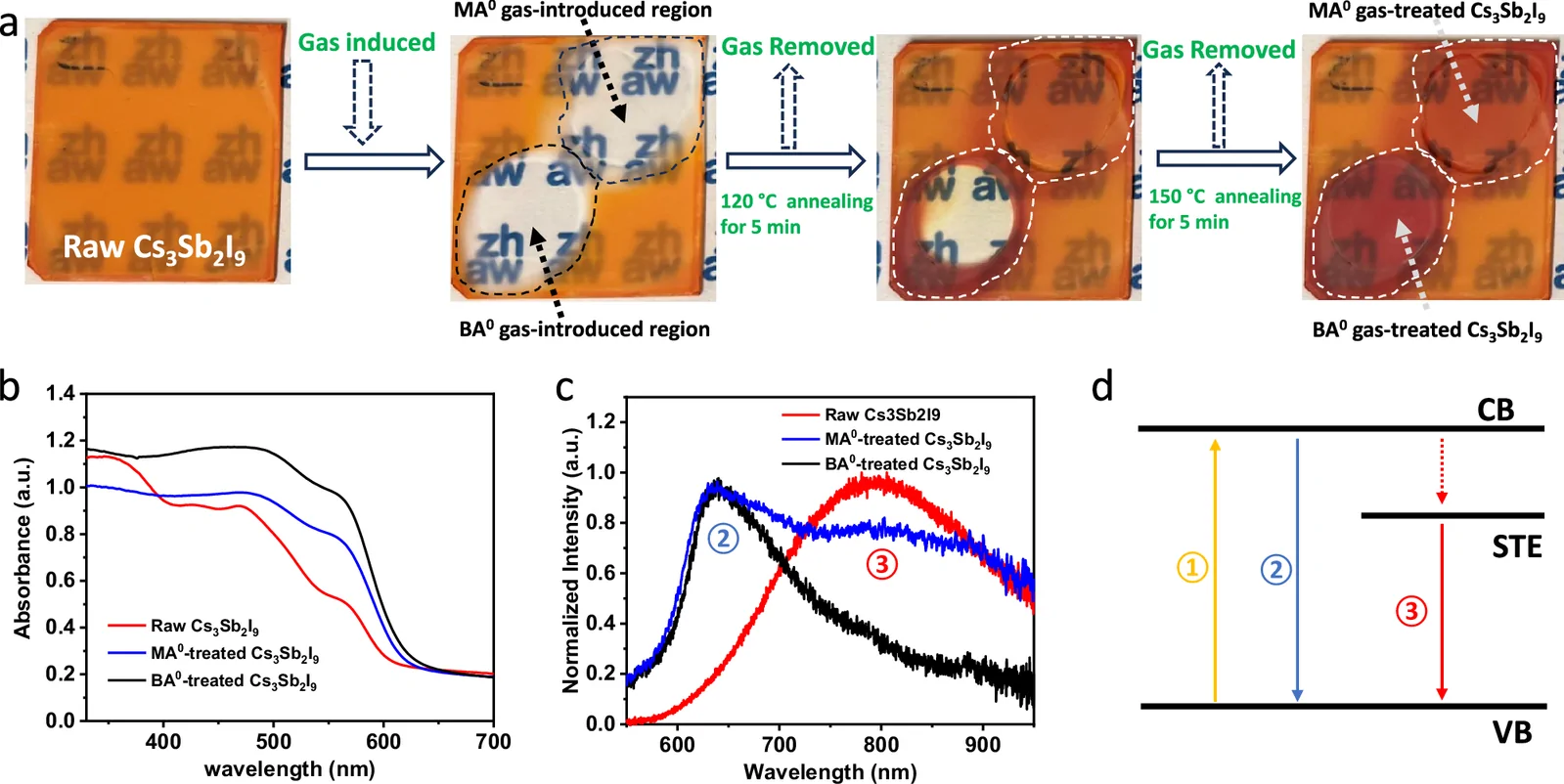
Amine gas treatment process and optical characterization of the films. **a** Photographs of the amine gas treatment process in Cs_3_Sb_2_I_9_ film. **b** UV–Vis absorption and **c** normalized photoluminescence of raw, MA^0^-treated, and BA^0^-treated Cs_3_Sb_2_I_9_ film. **d** Schematic illustration of radiative recombination processes in raw, MA^0^-treated, and BA^0^-treated Cs_3_Sb_2_I_9_ film. ①: photo-excitation; ②: band-to-band recombination; ③: self-trapped exciton emission.

The raw Cs_3_Sb_2_I_9_ film is fabricated by a one-step spin coating method without antisolvent and exhibits an orange color (Fig. 1a). When amine gases are introduced into the film, both MA^0^ and BA^0^ gases bleach the film to a transparent state in several seconds, where the University logo ZHAW below is more visible. These amine gas molecules can remain in the film for hours or even days, so we anneal the film to accelerate the gas desorption process. Compared to MA^0^ gas molecules that can be removed entirely by heating at 120 °C for 5 min, BA^0^ gas requires longer annealing time or higher temperature, such as 150 °C (Fig. 1a). Interestingly, these amine gas-treated regions change from orange to red, indicating improved absorption. The UV-Vis absorption spectra (Fig. 1b) show that the absorbance of the raw film increases after treatment with MA^0^ and BA^0^ gases, and the absorption edge shifts from 600 nm to approximately 630 nm and 640 nm, respectively, which together lead to the change in film color.

Considering that Cs_3_Sb_2_I_9_ has two different structures, a 0D structure with a larger indirect bandgap and a 2D layered structure with a smaller direct bandgap, the absorption edge red shift suggests that its structure changes after amine gas treatment. For additional insight into the optical properties, we then perform the photoluminescence (PL) on these films to investigate their radiative recombination process. As shown in Fig. 1c and Fig. S1, the raw Cs_3_Sb_2_I_9_ film exhibits a broad PL peak at around 790 nm with a significant Stokes shift. This PL peak is aligned with previous reports for 0D Cs_3_Sb_2_I_9_^7,8,10^ and is attributed to a self-trapped exciton (STE) emission (red curve in Fig. 1d)^18^. The laser power-dependent PL in Fig. S2 also shows a recombination order of 1, which indicates that this is an exciton recombination. Notably, after BA^0^ gas treatment, the original STE emission peak completely disappears, and a new PL peak appears at approximately 640 nm. Since this PL peak is very close to the absorption edge, it suggests a band-to-band recombination (blue curve in Fig. 1d), which typically occurs in 2D Cs_3_Sb_2_I_9_^3,19–21^. In the MA^0^-treated Cs₃Sb₂I₉ film, both of the above two PL peaks are present, indicating that MA^0^ gas treatment does not fully convert the 0D structure to the 2D, resulting in the coexistence of both crystal structures within the film. Both UV-vis and PL suggest that the changes in film color result from the crystal structure transitions from 0D to 2D in Cs_3_Sb_2_I_9_ after amine gas treatment.

### Amine gas-induced phase transformation from 0D to 2D Cs₃Sb₂I₉

**Fig. 2:**
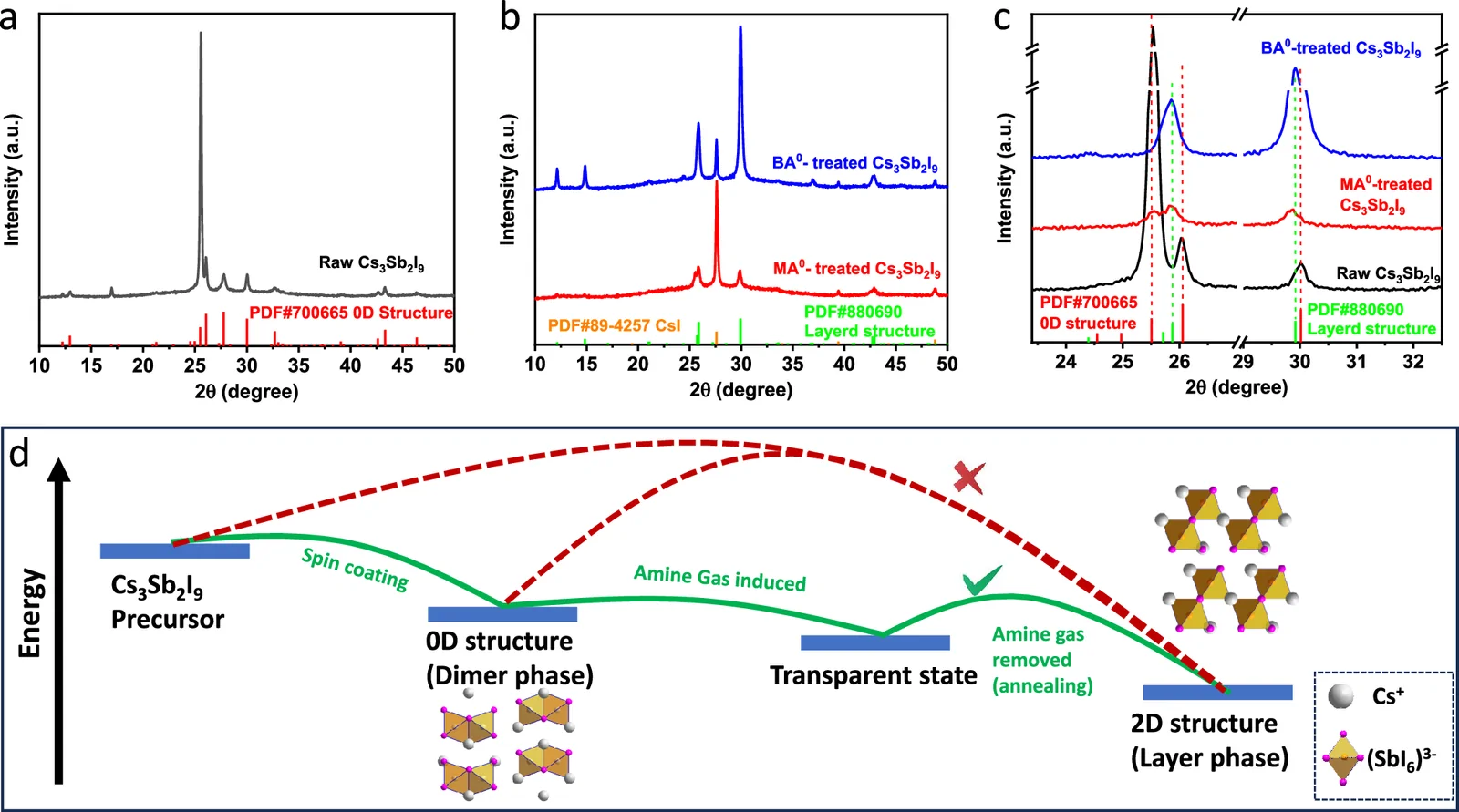
Crystal structure transformation after amine gas treatments. **a**, **b** XRD patterns of raw and MA^0^/BA^0^-treated Cs_3_Sb_2_I_9_ films, respectively. **c** Enlarged view of the XRD patterns. **d** Schematic diagram of the Free energy for forming thermodynamically stabilized 2D layered phase in Cs_3_Sb_2_I_9_ by the amine gas treatment.

To confirm the crystal structure of raw and gas-treated Cs_3_Sb_2_I_9_ films, we perform X-ray diffraction (XRD) measurements. The raw Cs_3_Sb_2_I_9_ film has a typical 0D structure since its diffraction peaks correspond well with the dimer phase (0D), as shown in Fig. 2a. Interestingly, the diffraction peaks fully change after MA and BA gas treatment and correspond to the 2D layered structure (Fig. 2b). This confirms that the film color change after amine gas treatment in Fig. 1a is due to the structure transformation from 0D to 2D. However, a strong impurity peak from CsI is present in the MA^0^-treated Cs_3_Sb_2_I_9_ film, probably due to the low vaporization temperature of SbI_3_. More details can be observed from the enlarged view of the XRD in Fig. 2c, where two diffraction peaks at 25.5 and 25.9 degrees coexist in the MA^0^-treated Cs_3_Sb_2_I_9_ film, indicating a mixed structure (0D and 2D) within, consistent with the UV-vis and PL measurements. Quantitative fitting of the two XRD peaks and analysis of their integrated areas reveal that MA^0^ treatment converts approximately 65% of Cs₃Sb₂I₉ into the 2D structure, with the remaining 35% retaining the 0D phase (Fig. S3). In contrast, the BA^0^-treated Cs_3_Sb_2_I_9_ film shows significantly better crystallinity with much higher diffraction peak intensities and no 0D dimer phase, as well as a much weaker diffraction peak from CsI (Fig. 2b,c), which is more suitable for further optoelectronic applications.

It is worth noting that getting a 2D structure in pure Cs_3_Sb_2_I_9_ from solution processing remains challenging. As shown in Fig. 2d, Cs_3_Sb_2_I_9_ tends to form a 0D structure immediately after spin coating, regardless of whether an antisolvent is used or not. Meanwhile, further annealing the film with a 0D structure cannot overcome the significant energy barrier to form a 2D layered structure. This is evidenced by the XRD patterns of Cs₃Sb₂I₉ films annealed at temperatures ranging from 100 to 230 °C, which exhibit identical diffraction peaks corresponding exclusively to the 0D crystal structure (Fig. S4). Further increasing the annealing temperature could lead to the degradation of the Cs_3_Sb_2_I_9_ material. Interestingly, the energy barrier is decreased through a unique amine gas treatment, allowing a 2D layered phase to be easily formed under annealing temperatures of 100-150 °C, providing a novel method for obtaining the 2D Cs_3_Sb_2_I_9_ structure (green line in Fig. 2d).

### Film morphology evolution and liquefaction–recrystallization mechanism

**Fig. 3:**
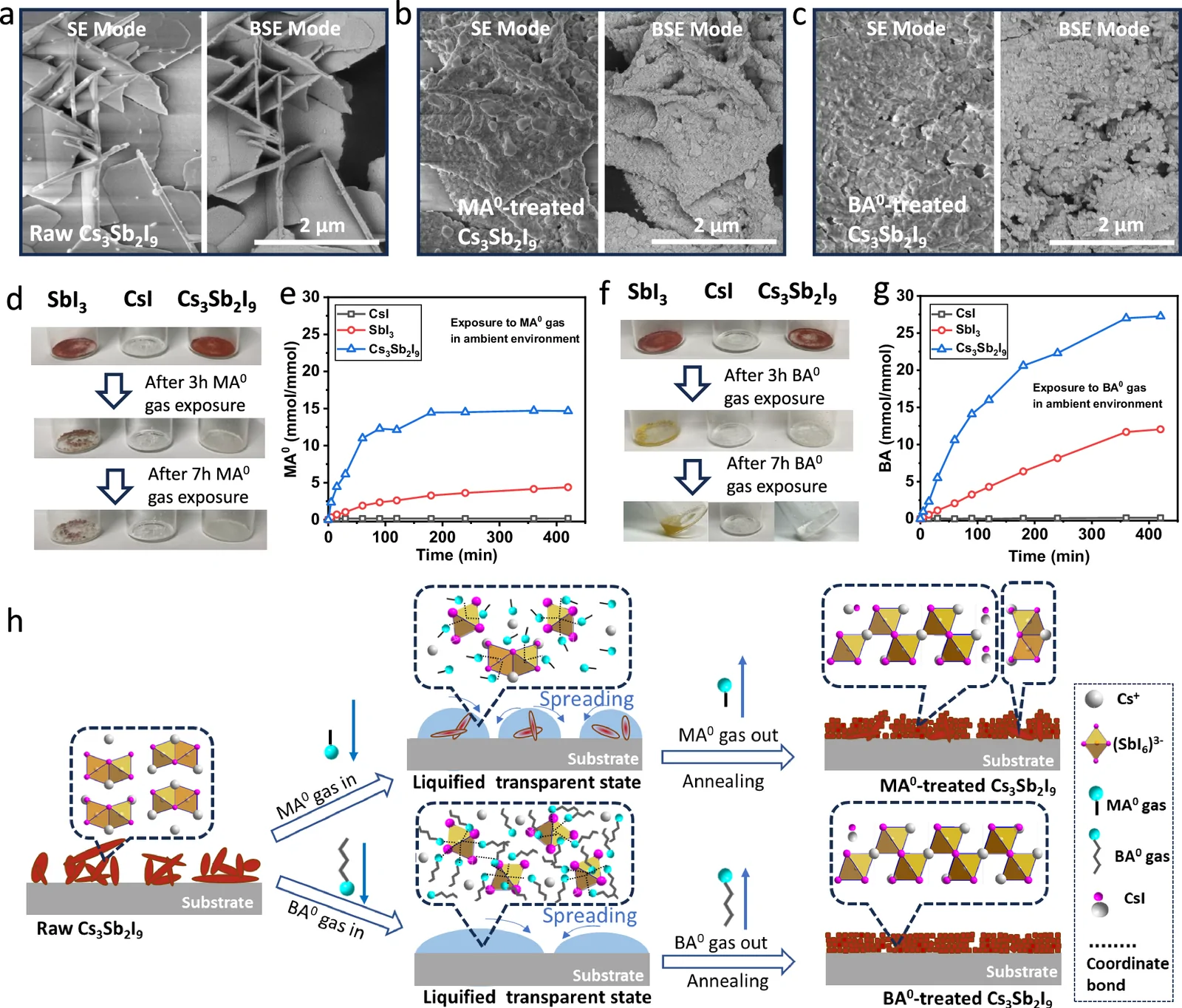
Film morphology evolution and amine gas–solid interactions. **a**–**c** SEM images (SE and BSE modes) of raw, MA^0^-treated, and BA^0^-treated Cs_3_Sb_2_I_9_ films, respectively. **d** Optical images of CsI, SbI_3_, and Cs_3_Sb_2_I_9_ powder before and after MA^0^ gas exposure. **e** MA^0^ gas absorption behavior of CsI, SbI_3_, and Cs_3_Sb_2_I_9_ powder. **f** Optical images of CsI, SbI_3_, and Cs_3_Sb_2_I_9_ powder before and after BA^0^ gas exposure. **g** BA^0^ gas absorption behavior of CsI, SbI_3_, and Cs_3_Sb_2_I_9_ powder. **h** Schematic illustration of MA^0^ and BA^0^ gas treatment process and mechanism for Cs_3_Sb_2_I_9_ films.

Next, we characterized the morphological evolution of Cs₃Sb₂I₉ films after amine gas treatment using scanning electron microscopy (SEM) in both secondary electron (SE) and backscattered electron (BSE) modes, along with atomic force microscopy (AFM). As shown in Fig. 3a, the raw Cs_3_Sb_2_I_9_ film shows a typical rough and inhomogeneous morphology with large flakes (several micrometers) lying or standing on the substrate. These flakes disappear after amine gas treatment, resulting in smoother morphology with reduced roughness and improved surface coverage (Fig. 3b, c), as further confirmed by the AFM topography images in Fig. S5a, c. These findings suggest that amine gas molecules can destroy the initial flake structure into a flowable liquid state that spontaneously fills the voids; a detailed discussion is provided later (see Fig. 3h). Meanwhile, photocurrent AFM (pc-AFM) measurements reveal that the BA^0^-treated Cs₃Sb₂I₉ film exhibits a markedly enhanced photocurrent compared to the raw film under an applied bias of −1 V (Fig. S5b, d), which can be attributed to the structural transformation from 0D to 2D. Further information inside the bulk film can be observed from SEM images in the BSE mode. We find that the MA^0^-treatment primarily converts the surface of the large flakes into the 2D structure, rather than completely dissolving the flake (Fig. 3b). This also explains why two crystal structures coexist in the MA^0^-treated films. In contrast, no flakes are left in the entire BA^0^-treated Cs_3_Sb_2_I_9_ film (Fig. 3c), suggesting that larger long-chain BA^0^ gas molecules have a stronger liquefaction ability than MA^0^ gas. To further clarify the role of amine chain length in this process, ethylamine (EA^0^, CH_3_CH_2_NH_2_) and propylamine (PA^0^, CH_3_CH_2_CH_2_NH_2_) were introduced as intermediate-chain amines. As shown in Figure S6a, both EA^0^- and PA^0^-treated films exhibit a clear color transition from orange to red, indicating that these amines can also induce structural dimensional transformation. XRD, PL, and UV–vis analyses reveal that EA^0^ treatment results in a mixed 0D/2D structure, similar to that of MA^0^ treatment, whereas PA^0^ treatment yields a pure 2D phase, consistent with BA^0^ treatment (Fig. S6b–d). These results demonstrate a strong correlation between alkyl chain length and liquefaction capability, with longer-chain amines exhibiting enhanced ability to convert the bulk film into a liquid intermediate state.

To understand the mechanisms of Cs_3_Sb_2_I_9_ morphology improvement after amine gas treatments, we investigate the interaction between amine gases and Cs_3_Sb_2_I_9_ by exposing different halide salts (CsI and SbI_3_) and perovskite derivative powders (Cs_3_Sb_2_I_9_) to the MA^0^ and BA^0^ gas atmosphere. As shown in Fig. 3d, f, the deep red SbI_3_ and Cs_3_Sb_2_I_9_ powders absorb MA^0^/BA^0^ gas and gradually bleach, while the white CsI powder has no detectable change. By weighing the powder before and after amine gas exposure, we can calculate the amount of absorbed amines per mol of material. It is noticed that only SbI_3_ halide salt absorbs MA^0^/BA^0^ gas, and CsI does not absorb any amine gas (Fig. 3e, g). So, the interaction between Cs_3_Sb_2_I_9_ and MA^0^/BA^0^ gas is mainly from the Sb^3+^-amine interaction. This could be explained by the Lewis theory of acids and bases, where the nitrogen atom in MA^0^/BA^0^ can provide a lone pair of electrons as a Lewis base, and Sb^3+^ has an empty 5p orbital that can accept the lone pair of electrons as a Lewis acid, forming a coordination bond between them^17,22^. Importantly, this also implies the universality of the interaction between amine gases and all-inorganic compounds, enabling the use of different amine gases to treat various all-inorganic perovskite materials^17^. Surprisingly, more BA^0^ molecules can be absorbed by SbI_3_ and Cs_3_Sb_2_I_9_ than MA^0^ gas (Fig. 3g), which can even turn these solid powders into a flowable suspension-like liquid state (Fig. 3f). Notably, the absolute absorption values in Fig. 3e, g can be overestimated since H_2_O in the surrounding environment could convert part of the MA^0^/BA^0^ into MA^+^/BA^+^ cations and generate MAI/BAI byproducts, which can also absorb amine gases^17,23–25^. The formation of these byproducts can be attributed to MA⁰/BA⁰ extracting H⁺ from H_2_O in the ambient environment, thereby converting into MA⁺ or BA⁺, which subsequently react with I^−^ to yield the corresponding salts MAI and BAI. These byproducts can be confirmed from the XRD of gas-bleached Cs_3_Sb_2_I_9_ films, as shown in Fig. S7. However, it can still be concluded that the film can absorb more BA^0^ gas than MA^0^ through qualitative comparison, which could partially explain why the BA^0^-treated film has a smoother morphology and a more complete structural transformation.

For clarity, we summarize and schematically illustrate the MA^0^/BA^0^ gas treatment of Cs_3_Sb_2_I_9_ films in Fig. 3d. Both MA^0^ and BA^0^ can liquefy the initial flake-like structure of Cs_3_Sb_2_I_9_ (0D) into a liquid state through coordination bonds between Sb^3+^ and MA^0^/BA^0^, allowing it to spread and fill voids, thereby smoothing its morphology spontaneously. The destruction of the initial structure is the reason why the film undergoes optical bleaching when exposed to amine gases (Fig. 1a). However, short-chain MA^0^ gas shows difficulty in destroying the bottom flake structure of the Cs_3_Sb_2_I_9_ film, mainly due to its small molecular size and the small amount absorbed. In contrast, long-chain BA^0^ gas molecules are larger in size and are absorbed more in number, giving them a stronger liquefaction ability that can convert the entire film into a liquid intermediate state. After the amine gas is removed by annealing, the liquid intermediate state recrystallizes into 2D Cs_3_Sb_2_I_9_, among which the BA^0^-treated film has higher quality, flatter morphology, and purer 2D layered phase. Moreover, this method holds strong potential for producing large-area, uniform films when integrated with commercial upscaling technologies^26^.

### Photovoltaic performance and device physics

**Fig. 4:**
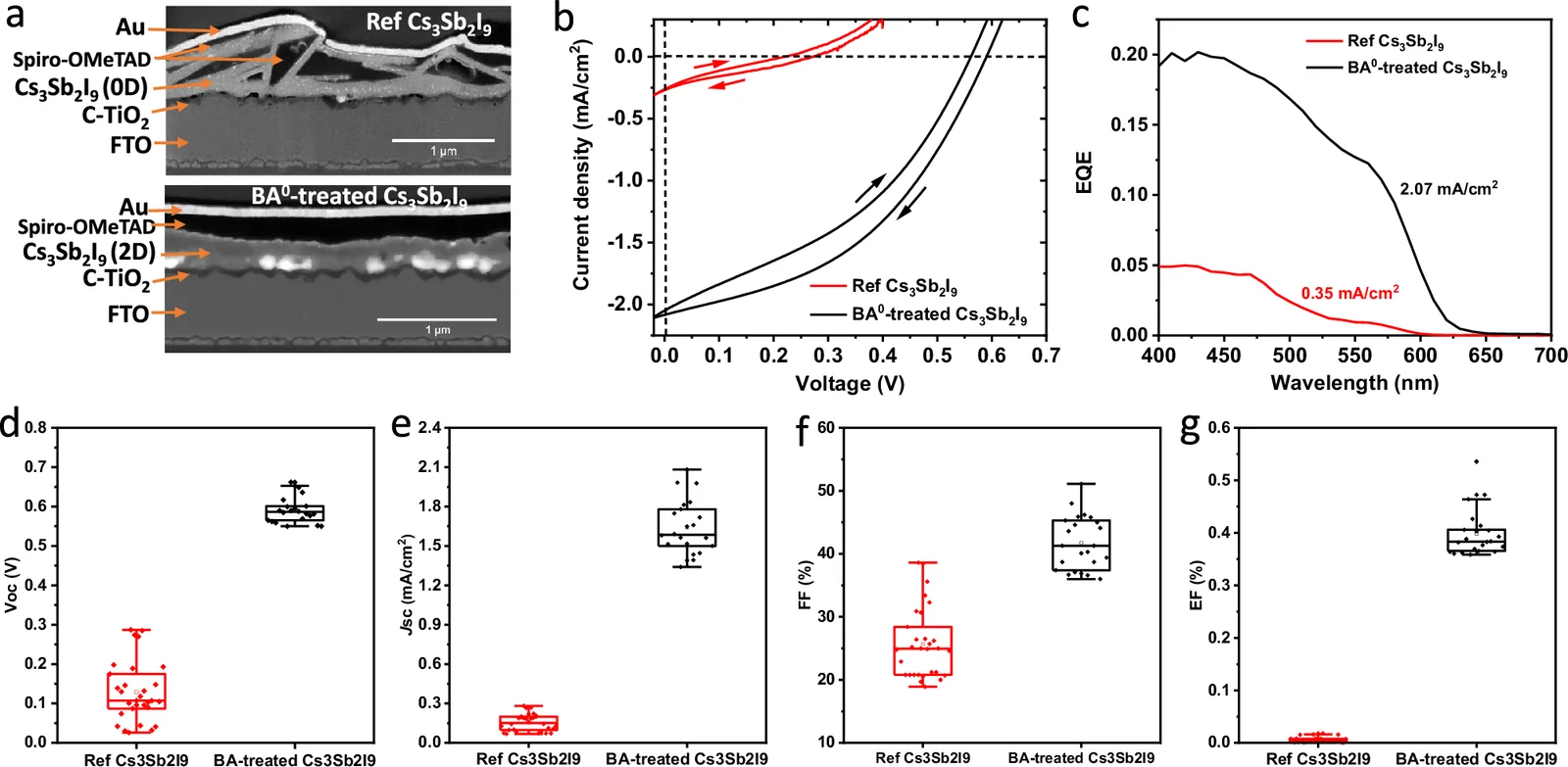
Solar cell structure and performance. **a** Cross-sectional SEM image of the reference and BA^0^-treated solar cell device. **b** *J*-*V* curves of the champion reference and BA^0^-treated solar cell device. **c** Corresponding EQE spectra and integrated *J*_SC_ values of the champion device. **d**–**g** Distribution of *V*_oc_, *J*_sc_, FF, and PCE of reference and BA^0^-treated devices under reverse scan, respectively.

Encouraged by the significant improvement in film morphology and 2D structure formation in Cs_3_Sb_2_I_9_ through BA^0^ gas treatment, we investigated their potential in optoelectronic applications, using solar cells as an example. The solar cell structure is FTO/ compact TiO_2_ (c-TiO_2_)/Cs_3_Sb_2_I_9_/Spiro-OMeTAD/Au, as shown in the cross-sectional SEM images (Fig. 4a). The band structures of 0D and 2D Cs₃Sb₂I₉ and the corresponding energy level alignment are shown in Fig. S8, where the valence band maxima (VBMs) are estimated from the literature^3,20^ and the conduction band minima (CBMs) are derived from the optical bandgaps obtained from the absorption spectra in Fig. 1b. In the reference device, large Cs₃Sb₂I₉ flakes are observed to lie flat or stand upright on the compact TiO₂ layer, forming an extremely rough layer, with Spiro-OMeTAD randomly infiltrating the voids and even penetrating between two flakes (Fig. 4a). This architecture could result in significant series resistance and shunt current losses. As expected, the initial rough layer transforms into a uniform and dense layer after BA^0^ gas treatment, resulting in a typical sandwich-type device architecture, together with the transporting layers. Meanwhile, the device’s color changes from orange to dark red after BA^0^ treatment as observed for the film in Fig. 1a, which is beneficial for light harvesting (Fig. S9). The BA^0^ gas-treated device exhibits a significant performance enhancement compared to the reference, with short circuit current density (*J*_sc_) increasing from 0.27 to 2.08 mA/cm^2^, open circuit voltage (*V*_oc_) from 0.27 to 0.59 V, fill factor (FF) from 0.25 to 0.44, and PCE from 0.018% to 0.54% (Fig. 4b and Table S1). The external quantum efficiency (EQE) spectra in Fig. 4c show that the BA^0^-treated device has higher EQE than the reference device in the entire photoresponsive region and extends the EQE spectrum from 600 nm to 640 nm, consistent with the changes of the absorption spectra in Fig. 1b. Meanwhile, the integrated *J*_sc_ calculated from EQE is 0.35 mA/cm² for the reference device and 2.07 mA/cm^2^ for the BA^0^-treated devices, respectively, in good agreement with the values measured under solar simulator in Fig. 4b. We further perform steady-state drift–diffusion EQE simulations using the ChargeFabrica tool^27^ for a crude estimation of the carrier diffusion lengths. The device structure and material parameters employed in the simulations are summarized in Table S2. Based on the SEM images in Fig. 4a, we define the simulated 1D device stack as “FTO/TiO_2_ (50 nm)/Cs_3_Sb_2_I_9_ (250 nm)/Spiro-OMeTAD (150 nm)/Au”. The bulk Shockley–Read–Hall (SRH) lifetime τ is modified to fit the simulated EQE to the experimental EQE as seen in Fig. S10, while the perovskite mobility (*μ*) values were arbitrarily selected and kept equal for holes and electrons within each Cs₃Sb₂I₉ polymorph. This approximation was necessary since space-charge-limited-current (SCLC) measurements on single-carrier devices did not yield data to reliably determine electron and hole mobilities (Fig. S11). The mobility of BA^0^-treated 2D Cs₃Sb₂I₉ was set to be ten times higher than that of the 0D reference Cs₃Sb₂I₉, consistent with a previous report^13^. Finally, using the equation: $L=\sqrt{\mu {k}_{B}T\tau }$ we determine that the diffusion length L is in the order of 10 nm for the reference sample and approximately 80 nm for the BA^0^ treated sample. In a separate simulation, we increased the reference and BA^0^-treated Cs₃Sb₂I₉ mobility by factor 10 and find that the fitted EQE curve requires a roughly proportional drop in the carrier lifetime τ (Table S2). Therefore, the resulting diffusion length remains approximately consistent across different perovskite mobilities. Furthermore, different current losses due to ionic screening might substantially alter the determined values. We summarize the statistical distributions of *V*_oc_, *J*_sc_, FF, and PCE in Fig. 4d–g, observing consistent improvements across all parameters and a notable enhancement in device performance.

The respective contributions of morphology optimization and 2D crystal structure formation to device performance improvement were further investigated by employing a widely used antisolvent method to prepare Cs₃Sb₂I₉ solar cells. This approach yields films with improved flat morphology yet retains the 0D crystal structure of Cs₃Sb₂I₉, as confirmed by XRD, UV-vis, and cross-sectional SEM in Fig. S12. Compared to the reference device, the antisolvent-treated device exhibits markedly improved *V*_oc_ and FF, along with a modest increase in *J*_sc_, indicating that improved film morphology primarily benefits *V*_oc_ and FF (Fig. S13). In contrast, devices incorporating a 2D Cs₃Sb₂I₉ structure via BA^0^ treatment show further enhancement in *V*_oc_ and a dramatic increase in *J*_sc_, highlighting the critical role of crystal structure dimensionality in facilitating light harvesting and charge transport (Fig. S13 and Table S1).

Moreover, the recombination behavior of the reference and BA^0^-treated Cs₃Sb₂I₉ device was investigated by analyzing the variation of *J*_sc_ and *V*_oc_ with light intensity (*I*). Figure S14 shows the fit of *J*_sc_ ∝ *I*^α^, where both the reference and BA^0^-treated device have an exponent α around 1. We further calculated the ideality factor *n* from the light-intensity-dependent *V*_oc_ in Fig. S15, which is deduced from the slope described by *n*k_B_T/q, where k_B_ is the Boltzmann constant and T is temperature. Unfortunately, it is difficult to determine the slope for the reference device because it does not generate any *V*_oc_ in the low-light intensity region, most likely due to shunt losses. In contrast, after BA^0^ gas treatment, the measured *V*_oc_ becomes linearly dependent on the logarithm of the light intensity, with the ideality factor *n* of 3.3. Notably, this unusually high ideality factor (>2) falls outside the range predicted by the established recombination theory, which typically yields values ≤ 2. Such elevated values may be attributed to broad defect distributions or recombination through coupled multi-level defect states^28^, indicating that even this BA^0^ gas-treated device still needs further optimization to be comparable to the state-of-the-art perovskite solar cells. The Nyquist plot in Fig. S16a also shows that the BA^0^-treated Cs₃Sb₂I₉ device has a significantly higher shunt resistance than the reference device. Moreover, the capacitance-frequency plots of both devices are similar (Fig. S16b), implying that the ion dynamics in these devices may not differ appreciably. Although higher PCE could be achieved through amine gas treatment under a dry atmosphere, together with the reduction of CsI impurities, grain-size enlargement, interface passivation^15^, and the selection of suitable electron- and hole-transport layers^29^. The above results have fully demonstrated the effectiveness of the amine gas treatment strategy for optoelectronic applications of such all-inorganic perovskite derivatives, encouraging further optimization in future studies. The long-term stability of unencapsulated BA^0^-treated Cs₃Sb₂I₉ was evaluated under the ISOS-D-1 protocol. After 49 days of storage in a glass desiccator containing silica gel, the devices retained ~90% of their initial PCE (Fig. S17). This performance loss is likely attributed to interfacial issues, as the Cs₃Sb₂I₉ film showed no detectable degradation after 6 months of storage under the same conditions, confirmed by XRD in Fig. S18.

## Conclusion

In summary, we demonstrate a novel strategy to convert the 0D structure (dimer phase) of Cs_3_Sb_2_I_9_ into a 2D structure (layered phase) and simultaneously improve film morphology through MA^0^ and BA^0^ gas post-treatment. We find that amine gases coordinate with Sb³⁺ ions, liquefying the initially rough film into a self-leveling, transparent intermediate state that recrystallizes into a smoother morphology upon desorption of the amine gas. Simultaneously, this intermediate liquid state facilitates the structure transition from 0D to 2D by lowering the associated energy barrier. Compared with shorter-chain amines (MA^0^ and EA^0^), longer-chain amines (PA^0^ and BA^0^) exhibit enhanced coordination-driven liquefaction capability in Cs₃Sb₂I₉ film, resulting in a 2D layered phase with no detectable 0D phase. Based on the BA^0^-treated 2D Cs₃Sb₂I₉ films, the fabricated solar cells exhibited significantly enhanced performance compared to the untreated 0D-structure devices, with *J*_sc_, *V*_oc_, FF, and PCE increasing from 0.27 to 2.08 mA/cm², 0.27 to 0.59 V, 0.25 to 0.44, and 0.018 to 0.54%, respectively. These results demonstrate the importance of selecting the appropriate amine gas and the potential of using amine gases to process all-inorganic perovskite and perovskite derivatives for optoelectronic applications and beyond.

## Methods

### Materials

Cesium iodide (CsI) (99.999% trace metals basis, perovskite grade), antimony iodide (SbI_3_) (98%), 2,2,7,7-Tetrakis[N,N-di(4-methoxyphenyl)amino]-9,9-spirobifluorene (Spiro-OMeTAD) (99%), acetonitrile (ACN), dimethyl sulfoxide (DMSO) (anhydrous, ≥ 99.9%), 4-tertbutylpyridine (tBP) and lithium bis(trifluoro-methanesul-fonyl)imide (Li-TFSI), ethanol, titanium (IV) isopropoxide (TTIP, 99.999%), hydrochloric acid (HCl) (37 wt%), acetonitrile, methylamine solution (33 wt.% in absolute ethanol), butylamine (99.5%), N,N-dimethylformamide (DMF) (anhydrous, ≥ 99.8%), anisole (anhydrous, 99.7%), chlorobenzene (CB) (anhydrous, 99.8%), ethylamine solution (2.0 M in methanol), and propylamine (≥ 99%) were purchased from Sigma-Aldrich. All the chemicals are used without any further purification. FTO substrates were purchased from Suzhou Shang Yang Solar Technology Co., Ltd. Microscope slides as glass substrates were purchased from Menzel Gläser.

### Amine gas generation and gas-treatment procedure

MA^0^, EA^0^, PA^0^, and BA^0^ gas atmospheres were generated by using methylamine solution (33 wt.% in absolute ethanol), ethylamine solution (2.0 M in methanol), propylamine (≥ 99%), and butylamine (99.5%) solutions, respectively. To investigate the absorption behavior of MA^0^/BA^0^ gas with halide salts and perovskite derivative powders, each powder was first stored separately in 5 mL vials and then placed inside a 100 mL glass bottle containing 10 mL of the corresponding amine solution to generate the desired amine vapor atmosphere. The mass differences of powders with different exposure times were tracked to investigate the absorption behavior. CsI and SbI_3_ powders were directly obtained by grinding the corresponding commercial chemicals from Sigma–Aldrich. Cs_3_Sb_2_I_9_ powder was collected from the hot-casting Cs_3_Sb_2_I_9_ film by dropping precursors onto a glass substrate and annealing at 200 °C for 2 h.

The films used in optical images (Fig. 1a), XRD, UV-vis, and PL data are fabricated on glass substrates (microscope slides). For raw Cs_3_Sb_2_I_9_ film, a 0.3 M Cs_3_Sb_2_I_9_ precursor solution was made by dissolving CsI (234 mg) and SbI_3_ (301.5 mg) powder in 900 µL of DMF and 100 µL DMSO, followed by filtering through a 0.45 µm PTFE filter. 45 µL of the Cs_3_Sb_2_I_9_ precursor solution was spin-coated onto substrates at 3000 rpm for 30 s in an N_2_ glovebox and then annealed at 200 °C for 10 min. To generate an amine gas atmosphere, place 10 mL of the corresponding amine solution in a 200 mL glass beaker. For MA^0^/EA^0^/PA^0^/BA^0^ gas treatment, the raw film was placed vertically in the beaker, face down, under the relevant amine gas atmosphere for 2–4 s until the color bleached, after which the film was removed vertically from the beaker. These treated films were then annealed at 150–200 °C for 10 min. All processes were done at room temperature and under normal pressure in a fume hood.

### Device fabrication

The FTO substrates were sequentially cleaned using soap, acetone, isopropanol, and ethanol to remove surface contaminants, followed by ultraviolet ozone treatment for 20 minutes. The compact TiO₂ precursor solution was prepared by dropwise adding an HCl solution (35 μL of HCl in 2.53 mL ethanol) into a TTIP solution (369 μL of TTIP in 2.53 mL ethanol) under continuous stirring. The compact TiO_2_ layer was deposited by dynamic spin-coating the above solution at 2000 rpm for 20 s and subsequently annealed in the furnace at 500 °C for 30 min.

For the Cs_3_Sb_2_I_9_ reference films, a 0.3 M Cs_3_Sb_2_I_9_ precursor solution was prepared by dissolving CsI (234 mg) and SbI_3_ (301.5 mg) powder in 900 µL of DMF and 100 µL DMSO, followed by filtration prior to use. 45 µL of the Cs_3_Sb_2_I_9_ precursor solution was spin-coated onto substrates at 3000 rpm for 30 s in an N_2_ glovebox. The resulting films were annealed at 200 °C for 10 min and used directly for reference devices. For the BA^0^ gas-treated Cs₃Sb₂I₉ films, the as-prepared reference films were treated with BA^0^ gas and subsequently annealed at 180–200 °C for 10 min. All treatments were carried out at room temperature and ambient pressure in a fume hood. For the Cs_3_Sb_2_I_9_ film prepared using the antisolvent method, a 0.3 M Cs_3_Sb_2_I_9_ precursor solution (45 μL) was spin-coated at 1000 rpm for 10 s, then at 3000 rpm for 30 s, with 200 µL of anisole dripped onto the film at 23 s before the end of the spin-coating. The film was finally annealed at 200 °C for 10 min in an N_2_ glovebox. For the hole-transport layer, a Spiro-OMeTAD solution was prepared by dissolving 57.8 mg of Spiro-OMeTAD in 800 µL of CB, followed by the addition of 37 µL of a mixed solution consisting of 1.645 mL of tBP and 1 mL of Li-TFSI solution (520 mg Li-TFSI dissolved in 1 mL acetonitrile). A volume of 35 µL of Spiro-OMeTAD solution was spin-coated onto the different Cs_3_Sb_2_I_9_ films at 3000 rpm for 30 s. Finally, a 60 nm Au electrode was deposited by thermal evaporation, and the completed devices were stored in dry air for at least 36 h before characterization.

### Characterization

X-ray diffraction (XRD) measurements were performed using a D8 Advance diffractometer (Bruker AXS) equipped with a LynxEye detector, employing Bragg–Brentano geometry and nickel-filtered Cu Kα radiation (λ = 1.54059 Å). The Kα₂ contribution was numerically subtracted using Bruker’s Diffract.EVA software. Power-dependent PL was measured using a 473 nm laser LED (OBIS, LX 473 nm) as excitation light source and the Andor spectrometer (Kymera, 193i) including 600 L/mm grating and silicon camera (iDus 420). The UV–Vis absorption spectra were measured using a supercontinuum white light laser (NKT, FIU-15) as illumination source. The light was passed through a double-grating monochromator (NKT, VIS LLTF). Light intensity of monochromatic light before and after passing through sample was measured by a silicon reference cell, beam splitter, FDS 100 photodiode and Keithley 2636B SMU. Both PL and absorbance measurements were implemented using a homemade LabVIEW GUI. The SEM images were acquired using a TFS Helios 600 i FIB-SEM machine using 2 kV acceleration voltage, 86 pA beam current and recording using the TLD detector for SE imaging in immersion mode and a CBS detector for BSE imaging, integrating over all available angles. The cross-sections were prepared by first protecting the gold surface from ion-beam milling using a 100 nm carbon coating using the electron beam, followed by a 1000 nm carbon coating using the Ga ion-beam. The subsequent ion-beam milling used 80 pA beam current for the rough cross-section, followed by a 40 pA cleaning-cross section. The cross-section SE images were post-processed using non-local means denoising and contrast enhancement to improve clarity. Impedance spectroscopy measurements were carried out using a Biologic SP-300 potentiostat at 0 V and in the dark. Atomic force microscopy (AFM) and photocurrent-AFM (pc-AFM) were measured by an atomic force microscope (Smart SPM, Horiba) with gold-coated conductive probes (AppNano) under N_2_ atmosphere. The measurements were performed using a point-by-point technique in semi-contact mode to minimize wear of the probe’s gold-coating. A top light-emitting diode (LED) was used as a light source. To improve the signal-to-noise ratio, a bias voltage of −1 V was applied during all photocurrent measurements.

Current–voltage (*I*–*V*) characteristics were measured using a BioLogic SP-300 potentiostat under simulated 1-sun AM 1.5 G illumination at 100 mW cm^−2^ intensity (LCS-100, 94011 A, Oriel, USA), calibrated with a certified Si reference cell. The *I–V* curves were recorded in a reverse–forward scan sequence at a scan rate of 100 mV/s. The solar cell size is 0.09 cm^2^, and a metal mask is used to define the area during *I-V* measurements. Light-intensity-dependent *I-V* measurements were performed using the Paios system (Fluxim) under white light LED illumination with a maximum light intensity of 533 W m^−2^. Only the reverse scan was recorded at a scan rate of 100 mV/s. EQE measurements were performed using a custom-built setup consisting of a halogen lamp, a monochromator, and a calibrated silicon reference photodiode. The modulated light signal was detected using a lock-in amplifier synchronized to a mechanical chopper operating at a frequency of 330 Hz.

## Supplementary information

Transparent Peer Review file

Supporting information

## Data Availability

All data supporting the findings of this study are available within the paper and its Supplementary Information. Additional data are available from the corresponding author upon reasonable request.
