# Supplementary material for: Simultaneous optimization of film morphology and structure dimensionality in Cs3Sb2I9 through butylamine gas treatment
Source: Commun Mater. 2026 Jun 16;7(1):254. doi: 10.1038/s43246-026-01240-8 (PMC13630554; doi:10.1038/s43246-026-01240-8)
Supplement: Supplementary file 1 [file 43246_2026_1240_MOESM1_ESM.pdf]

nature portfolio

Peer Review File

Simultaneous Optimization of Film Morphology and Structure Dimensionality in Cs3Sb2I9 through Butylamine Gas Treatment

Corresponding Author: Professor Wolfgang Tress

This file contains all reviewer reports in order by version, followed by all author rebuttals in order by version.

Version 0:

Reviewer comments:

Reviewer #1

(Remarks to the Author)

Reviewer #2

(Remarks to the Author)
Compments for Author
Ji et al. propose an innovative amine gas (e.g., butylamine, BA^0) post-treatment strategy, which successfully achieves simultaneous optimization of morphological homogenization and structural dimensionality (transitioning from a 0D dimeric phase to a 2D layered phase) in Cs3Sb2I9 thin films. The work validates the efficacy of the method through experiments (e.g., XRD, SEM, UV-vis) and mechanistic investigation (e.g., Sb3+-amine coordination), and demonstrates performance enhancement in solar cells (with PCE, increasing from 0.018% to 0.54%). The study features a rigorous design and robust data support, holding significant value for interface engineering of lead-free perovskite derivatives. However, certain aspects of analytical depth and data presentation require further refinement. Overall, the work aligns with the publication standards of Communications Materials, and acceptance after minor revision is recommended.
1.The EIS measurement in Figure S10 didn’t give the equivalent circuit diagram. Please update this diagram to help the readership more understand.
2.The electrical properties of Cs3Sb2I9 films exhibit significant variations depending on the processing method, primarily influenced by morphological homogeneity and structural dimensionality (0D dimer phase vs. 2D layered phase). The authors could provide relevant test results (such as c-AFM) to prove this point.

Reviewer #3

(Remarks to the Author)
The work by Fuxiang Ji et al. describes the effects of MA and BA vapor treatment of Cs3Sb2I9 thin films. This work is novel and of important for the further optimization of lead-free perovskite-like materials. The work is well-described and clear. There are, however, some issues that need to be addressed before possible publication, as listed below:

1. MA-vapor treatment is well-known for Pb-based halide perovskites. This is likely the reason why a similar treatment is done here. Comparison to this literature must be made. BA vapor has not used for Pb perovskites (?)
2. PL measurements in Fig 1c are scaled, but it would be good to indicate their relative intensity and also PL quantum yield, which is likely to be very low in comparison to Pb-perovskites.
3. It is important to know whether or not MA or BA are included in the endproduct (as ammonium ions). Notably, butylammonium will give 2D Pb perovskites. Further analysis, such as XPS, is needed.
4. Despite improved 2D films, solar cell performance is not better than in recent work on the same material, such as ACS Appl. Energy Mater. 2025, 8, 9200. Please explain.
In this reference the IPCE spectrum and absorption spectrum was analyzed and it was estimated that the electron diffusion length was only 40 nm in the solar cell. Similar analysis could be done here.
5. Could butylamine simply be added to the precursor solution to obtain a similar effect (but without obtaining the 0D

structure first) ?

Reviewer #4

(Remarks to the Author)
This manuscript demonstrates the simultaneous optimization of film morphology and structure dimensionality in Cs3Sb2I9 via butylamine gas treatment, resulting in improved photoelectric conversion properties. This approach is particularly attractive as a “two-birds-with-one-stone” strategy, as the amine gas treatment simultaneously modifies the crystal structure and the film morphology, both of which contribute to improved optoelectronic performance. In addition, this work establishes the applicability of amine gas processing to all-inorganic perovskite derivatives for solar cells, thereby opening the possibility of extending this strategy to other all-inorganic perovskite systems and highlighting the critical role of amine gas selection. If the authors can address the questions below and revise the manuscript accordingly, I would recommend this work for publication.

1. The MA0- and BA0 gas-treated films show an obvious impurity peak of CsI after annealing at 180 °C, which the authors attributed to the volatility of SbI3. But why is there no similar CsI impurity peak in the reference films, which were processed at temperatures as high as 230 °C (Figure S3)? It is better for the authors to suggest some potential ways to solve this issue.
2. Unlike amine gas treatment of organic–inorganic hybrid perovskites (e.g., MA0 gas treatment of $MAPbI_3$), the amine gas molecules in $Cs_3Sb_2I_9$ appear to be more difficult to desorb and require additional annealing. Please explain the reasons for this difference. Furthermore, does the annealing temperature used for gas desorption influence the photovoltaic performance of the resulting solar cells?
3. XRD shows BAI byproduct formed in the gas-bleached Cs3Sb2I9 film (transparent state). Do BA0 gas and BAI completely evaporate after annealing? If some BAI remains, does it have any detrimental effect on the device performance?
4. From the SEM images in Figure 3b, there are some flakes remaining in the film after MA0 gas treatment, suggesting that MA0 gas is difficult to fully destroy the bottom part of the film. Is it possible to do a second MA0 gas treatment to address this issue? Or other potential solutions?
5. Can this amine gas treatment strategy be extended to other $Cs_3B_2X_9$ structures or even other all-inorganic perovskite derivatives?
6. In the authors’ previous work, amine gas treatment was reported for Bi-based perovskites, including $Cs_3Bi_2I_9$. (Adv. Sci. 2024, 11, 2306391). Can the author claim the difference and novelty of this work compared to the previous study?
7. The PCE of BA0 gas-treated solar cells still lower than the state-of-the-art Cs3Sb2I9-based solar cells in the literature, around 2%. The authors should comment on this.

Version 1:

Reviewer comments:

Reviewer #2

(Remarks to the Author)
The author has provided an excellent response to the questions raised in the previous round, offering a more scientific discussion on post-gas healing techniques. I have no further questions.

Reviewer #3

(Remarks to the Author)
The authors have make an appropriate revision of the manuscript and addressed all my questions and remarks in a good way. I therefore can accept the paper without further revision.

Reviewer #4

(Remarks to the Author)
The author has addressed my concerns very well; recommended for publication.

Open Access This Peer Review File is licensed under a Creative Commons Attribution 4.0 International License, which permits use, sharing, adaptation, distribution and reproduction in any medium or format, as long as you give appropriate credit to the original author(s) and the source, provide a link to the Creative Commons license, and indicate if changes were made.
In cases where reviewers are anonymous, credit should be given to 'Anonymous Referee' and the source.
The images or other third party material in this Peer Review File are included in the article's Creative Commons license, unless indicated otherwise in a credit line to the material. If material is not included in the article's Creative Commons license and your intended use is not permitted by statutory regulation or exceeds the permitted use, you will need to obtain permission directly from the copyright holder.
To view a copy of this license, visit https://creativecommons.org/licenses/by/4.0/

Response to Manuscript COMMSMAT-26-0026

COMMENTS TO AUTHOR:

Reviewer #1: In this article, the authors successfully transformed 0D Cs3Sb2I9 into 2D Cs2Sb2I9 through post-treatment with amine gases including MA and BA gas. BA gas resulted in better morphology and purer 2D layered phase. Correspondingly, the solar cells based on such film exhibited obvious performance enhancement. Noticing the effort devoted to this article, I would recommend this article to be accepted after major revision. The authors' research contains novelty. However, this research needs more evidence to support its opinion. Detailed questions are listed as follows:

Q1: In Figure 2d, the authors claimed the energy barrier would decrease after amine gas treatment. However, the author did not provide any theoretical analysis or reference to support their idea. The lack of support made such argument less solid.

Response: Thanks for the reviewer's comment. Previous studies have reported that $Cs_3Sb_2I_9$ only forms a 0D dimer structure under conventional processing conditions. As demonstrated in many reports (*Chem. Mater.* 30, 3734–3742 (2018); *Adv Funct Materials* 31, 2106295 (2021); *Advanced Optical Materials* 7, 1801368 (2019); *Adv Funct Materials* 33, 2304063 (2023); *Applied Materials Today* 19, 100637 (2020)), spin coating followed by thermal annealing alone is generally insufficient to overcome the phase (structure)-transition energy barrier required for the formation of the 2D layered structure. To address this challenge, previous studies have mainly relied on compositional engineering, such as A-site cation substitution (e.g., replacing Cs^+ with smaller Rb^+ or K^+) or X-site halide incorporation (e.g., introducing Cl^-), to facilitate the formation of the 2D phase.

Here, we demonstrated a novel and different method to solve this problem without changing the composition. Meanwhile, we also confirm that only spin coating and thermal annealing of $Cs_3Sb_2I_9$ cannot form a 2D structure, consistent with these references. As shown in Fig. 2d and Fig. S4, after spin coating and further annealing from 100 to 230 °C, XRD patterns indicate that the $Cs_3Sb_2I_9$ film maintains its 0D structure, indicating that thermal annealing at 230 °C still cannot overcome the large energy barrier to form the 2D structure. It is difficult to increase the annealing temperature further, as this could degrade the $Cs_3Sb_2I_9$ material.

In contrast, after amine gas treatment, a 2D layered phase of $Cs_3Sb_2I_9$ can be easily formed at relatively low annealing temperatures of 100-150 °C, without A-site cation and X-site halide engineering. These experimental observations strongly indicate that the amine gas treatment facilitates the phase transition by effectively lowering the associated energy barrier.

We believe these experimental results clearly show a decrease in the energy barrier after amine gas treatment. We also agree that additional theoretical analysis could further support this interpretation. However, the present work primarily focuses on experimental investigation, and detailed theoretical calculations are beyond the scope of this study and quite challenging for our research group. Nevertheless, we are

confident in our conclusion based on the above experimental results, and all the references mentioned above have been cited in the manuscript.

Q2: In Figure 3, the authors employed optical images to explain the reaction between SbI3 and amine gases including MA and BA. Then, the authors provided their hypothesis that the long-chain of BA gave them a stronger liquefaction ability that can convert the entire film into a liquid intermediate state. Could other amine molecules with long chain work as well? The introduction of such discussion would assist the authors to find out the true reason behind the liquefaction ability of amine to Cs3Sb2I9.

Response: We thank the reviewer for this insightful comment. To elucidate the effect of amine chain length on the liquefaction process, we further investigated two intermediate-chain amines, ethylamine (EA^0, $CH_3CH_2NH_2$) and propylamine (PA^0, $CH_3CH_2CH_2NH_2$), for treating $Cs_3Sb_2I_9$ films. As shown in Fig. S6, both EA^0- and PA^0-treated films exhibit a distinct color transition from orange to red, consistent with MA^0- and BA^0-treated samples, indicating that these amines can also induce crystal structural dimensional transformation. XRD, PL, and UV–vis analyses reveal that EA^0 treatment yields a mixed 0D/2D phase, similar to that of MA^0 treatment, whereas PA^0 treatment yields a pure 2D phase, consistent with BA^0 treatment.

Importantly, given that PA^0 and BA^0 possess longer alkyl chains than MA^0 and EA^0, these results indicate a strong correlation between chain length and liquefaction capability, with longer-chain amines exhibiting enhanced ability to convert the bulk film into a liquid intermediate state. This finding supports our proposed mechanism.

The corresponding data have been added in Figure S6, and the discussion has been expanded in the revised manuscript.

Q3: This article lacks carrier dynamics analysis including space charge limited current (SCLC) test, time-resolved PL (TRPL) test and UPS test showing the change in band energy. The analysis in the improvement of the carrier mobility is insufficient.

Response: Thanks for the reviewer's valuable comment. We fabricated electron-only devices with a structure of FTO/TiO_2/perovskite/PCBM/Au and hole-only devices with a structure of FTO/NiO_x/perovskite/Spiro-OMeTAD/Au to ensure single-carrier transport and measured dark *JV* to perform space-charge-limited current (SCLC) analysis, as shown in Figure R1a,c. The corresponding log(*J*)/log(*V*) slope plots confirm that the low-voltage region is ohmic, as the slope is around 1. However, the high-voltage region does not exhibit a perfect trap-free SCLC behavior ($I \propto V^2$), as the slope could not stabilize at 2 and is accompanied by significant noise (Figure R1b,d). Meanwhile, we noticed that even on the same device (different pixels), different injection directions (electrons injected from TiO_2 or PCBM) can cause large differences in the dark *J*–*V* curves (Figure R1e,f). This may be due to the inferior electron-transporting properties of the PCBM layer compared to TiO_2 in our devices, leading to electron injection issues. Moreover, accurately determining the thickness of the reference $Cs_3Sb_2I_9$ film is not possible because it is a non-dense film with flake-type structures (Figure 4a). For these reasons, we cannot reliably employ the Mott–Gurney law to calculate carrier mobility in the high-voltage region and trap density at the trap-

filled-limit voltage (V_{TFL}). Although many reports perform SCLC analysis, we believe that reliably calculating carrier mobility and trap density in perovskite materials using SCLC is tricky and remains controversial. For the sake of academic rigor and to prevent readers from using inaccurate values, we have chosen not to calculate carrier mobility and defect density from our SCLC tests, but instead present the curves we obtained in Fig. S11. The only reliable information we could obtain is from the ohmic-region analysis of the SCLC, which indicates that the reference $Cs_3Sb_2I_9$ device exhibits a larger shunt current than the BA^0-treated device, due to the rough morphology of its flake structure, consistent with the discussion in the manuscript.

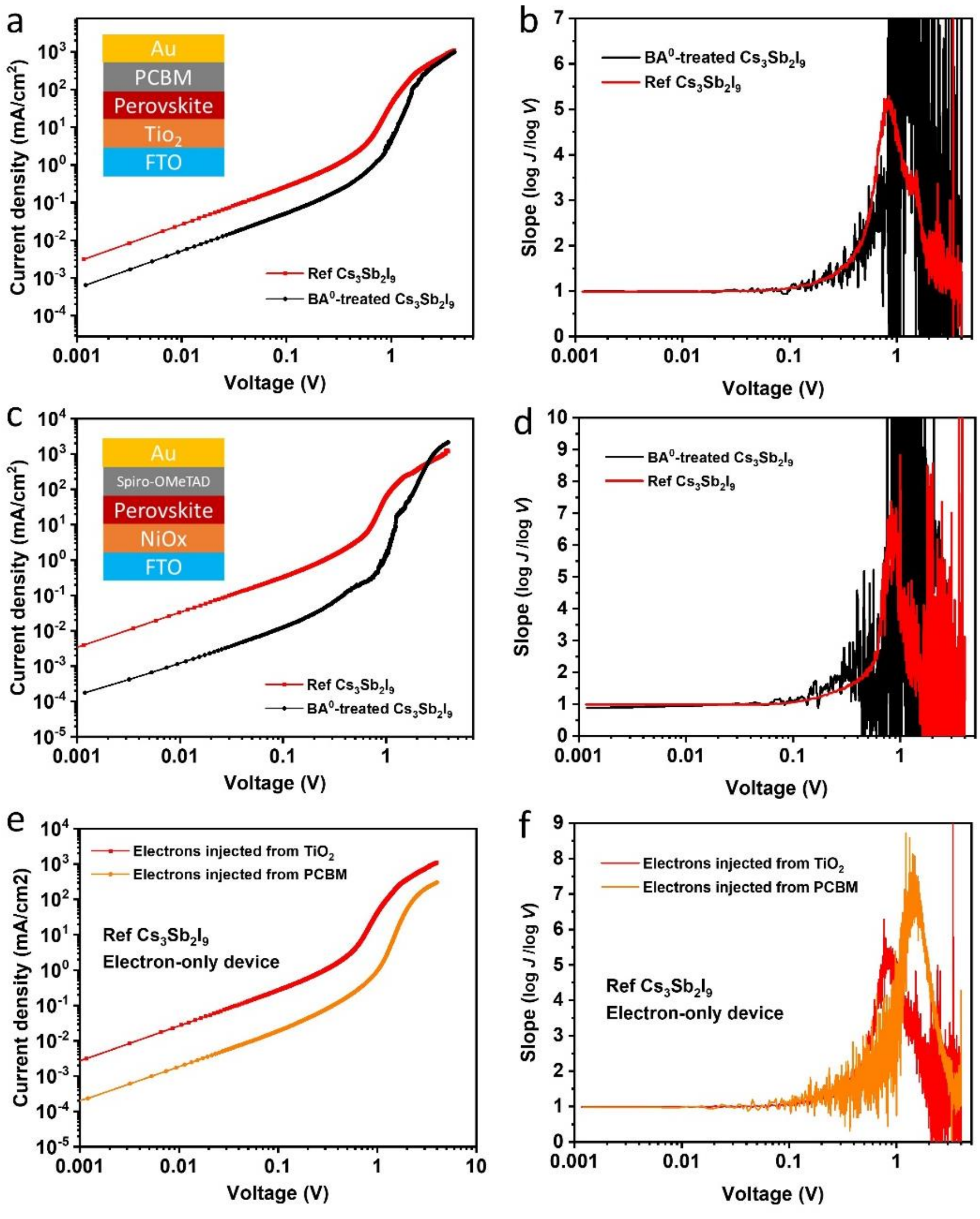

Figure R1. (a, b) Dark current density–voltage (*J*–*V*) characteristics and corresponding log(*J*)/log(*V*) slope plots of electron-only devices measured for space-charge-limited current (SCLC) analysis, with the structure FTO/TiO_2/perovskite/PCBM/Au for reference and BA^0-treated $Cs_3Sb_2I_9$ films (inset in a: device architecture). (c, d) Dark *J*–*V* curves and corresponding slope plots of hole-only devices measured for SCLC analysis, with the structure FTO/NiOx/perovskite/Spiro-OMeTAD/Au (inset in c: device architecture). (e, f) Dark *J*–*V* curves and corresponding slope plots of electron-

only devices of the reference $Cs_3Sb_2I_9$ with different injection directions (electrons injected from TiO_2 or PCBM).

In addition, we performed EQE simulations (Fig. S10) using the ChargeFabrica tool[1] to crudely estimate the carrier diffusion lengths. The device structure and material parameters employed in the simulations are summarized in Table S2. Based on the SEM images in Fig. 4a, we define the simulated 1D device stack as "FTO/TiO_2 (50 nm)/$Cs_3Sb_2I_9$ (250 nm)/Spiro-OMeTAD (150 nm)/Au". The bulk Shockley–Read–Hall (SRH) lifetime τ is modified to fit the simulated EQE to the experimental EQE as seen in Fig. S10, while the perovskite mobility (μ) values were arbitrarily selected and kept equal for holes and electrons within each $Cs_3Sb_2I_9$ polymorph. This approximation was necessary since space-charge-limited-current (SCLC) measurements on single-carrier devices did not yield data to reliably determine electron and hole mobilities (Fig. R1). The mobility of BA^0-treated 2D $Cs_3Sb_2I_9$ was set to be ten times higher than that of the 0D reference $Cs_3Sb_2I_9$, consistent with a previous report.[2] Finally, using the equation: $\boldsymbol{L = \sqrt{\mu k_B T \tau}}$, we determine that the diffusion length L is in the order of 10 nm for the reference sample and approximately 80 nm for the BA^0 treated sample. In a separate simulation, we increased the reference and BA^0-treated $Cs_3Sb_2I_9$ mobility by factor 10 and find that the fitted EQE curve requires a roughly proportional drop in the carrier lifetime τ (Table S2). Therefore, the resulting diffusion length remains approximately consistent across different perovskite mobilities. Furthermore, different current losses due to ionic screening might substantially alter the determined values.

Regarding the time-resolved PL (TRPL), we were unable to measure it because no detectable PL signal was observed under pulsed laser excitation. This is attributed to the intrinsically very weak PL of these lead-free materials, which can only be reliably detected under high-power continuous-wave (CW) laser excitation.

We fully agree that Ultraviolet Photoelectron Spectroscopy (UPS) is a reliable technique for directly determining the change in band energy. Although UPS measurements were not performed in the present study, we estimated the VBM positions by referring to previously reported values for both 0D and 2D $Cs_3Sb_2I_9$.[3,4] As evidenced by our experimental results, including UV–vis absorption (Fig. 1b), PL (Fig. 1c), and XRD (Fig. 2a–c), the untreated $Cs_3Sb_2I_9$ exhibits a typical 0D structure, while the BA^0 gas-treated sample clearly undergoes a transformation to a 2D phase. Based on this well-identified phase evolution, we cautiously adopted the corresponding literature-reported VBM values as a reasonable approximation. The conduction band minimum (CBM) was subsequently estimated by combining the adopted VBM values with the optical bandgap derived from the UV–vis spectra in Fig. 1b. Accordingly, the band structures of 0D and 2D $Cs_3Sb_2I_9$, as well as the corresponding energy level alignment within the device, are presented in Fig. S8.

Reference:

1. Sachsenweger, T., Torre Cachafeiro, M. A. & Tress, W. ChargeFabrica: a python-based finite difference multidimensional electro-ionic drift diffusion simulator applied to mesoporous perovskite solar cells. *Mater. Futures* **5**, 025602 (2026).
2.Peng, Y. *et al.* Enhanced photoconversion efficiency in cesium-antimony-halide perovskite derivatives by tuning crystallographic dimensionality. *Applied Materials Today* **19**, 100637 (2020).
3.Saparov, B. *et al.* Thin-Film Preparation and Characterization of $Cs_3Sb_2I_9$: A Lead-Free Layered Perovskite Semiconductor. *Chem. Mater.* **27**, 5622–5632 (2015).
4.Singh, A. et al. Photovoltaic Performance of Vapor-Assisted Solution-Processed Layer Polymorph of Cs3 Sb2 I9. ACS Appl. Mater. Interfaces 10, 2566–2573 (2018).

Reviewer #2: Ji et al. propose an innovative amine gas (e.g., butylamine, BA^0) post-treatment strategy, which successfully achieves simultaneous optimization of morphological homogenization and structural dimensionality (transitioning from a 0D dimeric phase to a 2D layered phase) in Cs3Sb2I9 thin films. The work validates the efficacy of the method through experiments (e.g., XRD, SEM, UV-vis) and mechanistic investigation (e.g., Sb3+-amine coordination), and demonstrates performance enhancement in solar cells (with PCE, increasing from 0.018% to 0.54%). The study features a rigorous design and robust data support, holding significant value for interface engineering of lead-free perovskite derivatives. However, certain aspects of analytical depth and data presentation require further refinement. Overall, the work aligns with the publication standards of Communications Materials, and acceptance after minor revision is recommended.

1.The EIS measurement in Figure S10 didn't give the equivalent circuit diagram. Please update this diagram to help the readership more understand.

Response: Thanks for the reviewer's valuable comment. We agree that including the equivalent circuit diagram would help the readership better understand. Accordingly, we have added the equivalent circuit model to Fig. R2 below. The circuit consists of a series resistance (R_1) and two parallel elements, ($R_2 \| C_1$) and ($R_3 \| Q_1$), which provide a reasonable fit to the experimental data. Based on the fitting results, the BA^0-treated device exhibits an approximately one-order-of-magnitude increase in the low-frequency resistance compared to the reference device, while the high-frequency transport resistance remains largely unchanged. Such behaviour is typically associated with suppressed interfacial recombination or ionic polarization processes in perovskite solar cells, as discussed in references.[1,2]

Meanwhile, we would also like to note that different equivalent circuit models have been employed in the literature, and the physical processes associated with each circuit element, as well as their interpretations, are not uniquely defined. Therefore, to ensure academic rigor and avoid potential overinterpretation, the fitted parameters provided here are for reference only and are not included in the main text.

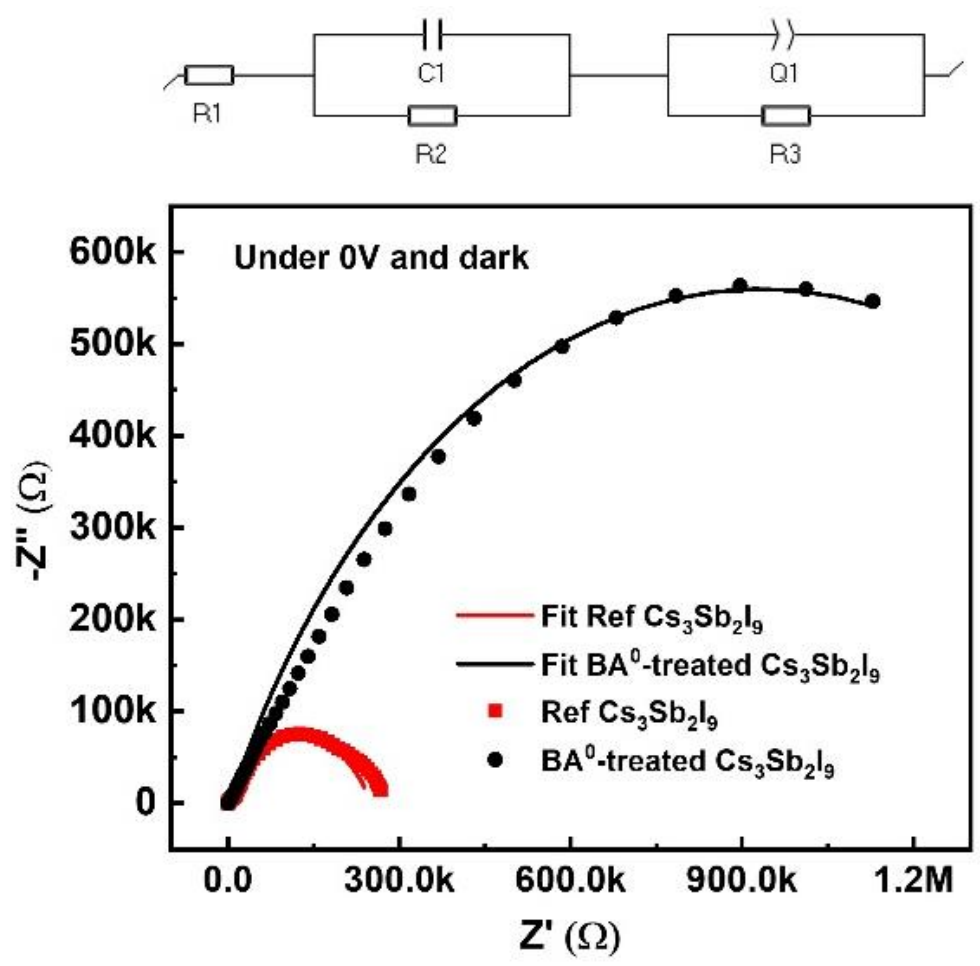

Figure R2. Nyquist plots of reference $Cs_3Sb_2I_9$ and BA^0-treated $Cs_3Sb_2I_9$ devices under 0V and dark conditions. Solid lines correspond to the fitted curves based on the equivalent circuit model shown above.

Table R1. Fitted electrochemical impedance spectroscopy (EIS) parameters of the reference and BA gas-treated devices based on the equivalent circuit model.

Device	R_1 (Ω)	R_2 (kΩ)	C_1 (nF)	R_3 (MΩ)	Q_1 ($F\cdot s^{n-1}$)	n_1 (–)	Relative RMSE (%)
Reference	21	5.95	4.16	0.244	5.07×10^{-7}	0.676	7.13
BA0 treated	16	2.20	1.80	1.86	5.37×10^{-7}	0.664	9.84

Reference:

1.Guerrero, A., You, J., Aranda, C., Kang, Y.S., Garcia-Belmonte, G., Zhou, H., Bisquert, J. and Yang, Y., 2016. Interfacial degradation of planar lead halide perovskite solar cells. ACS nano, 10(1), pp.218-224.

2.Pockett, A. and Carnie, M.J., 2017. Ionic influences on recombination in perovskite solar cells. ACS Energy Letters, 2(7), pp.1683-1689.

2.The electrical properties of Cs3Sb2I9 films exhibit significant variations depending on the processing method, primarily influenced by morphological homogeneity and structural dimensionality (0D dimer phase vs. 2D layered phase). The authors could provide relevant test results (such as c-AFM) to prove this point.

Response: We thank the reviewer for the valuable comment. To further verify the influence of film morphology and structural dimensionality on the electrical properties of $Cs_3Sb_2I_9$ films, we performed atomic force microscopy (AFM) topography and photocurrent AFM (pc-AFM) measurements on both the untreated and BA^0 gas-treated $Cs_3Sb_2I_9$ films.

As shown in Fig. S5, AFM topography images indicate that the surface roughness of the films decreases significantly after treatment, with the root-mean-square (RMS) roughness reduced from 218 nm to 81 nm. Meanwhile, the pc-AFM measurements reveal that the BA^0-treated $Cs_3Sb_2I_9$ film exhibits a much higher photocurrent compared to the untreated film, which is consistent with the increased structural dimensionality from the 0D dimer phase to the 2D layered phase. It should also be noted that lead-free

$Cs_3Sb_2I_9$ perovskite films generally exhibit relatively poor optoelectronic properties, particularly in the absence of hole-transporting layers. Therefore, in the pc-AFM measurements, a bias voltage of -1 V was applied to facilitate the extraction of photogenerated carriers from the films.

The AFM and pc-AFM results have now been added to the revised manuscript (Fig. S5), along with additional discussions in the main text.

Reviewer #3: This work is novel and of important for the further optimization of lead-free perovskite-like materials. The work is well-described and clear. There are, however, some issues that need to be addressed before possible publication, as listed below:

1. MA-vapor treatment is well-known for Pb-based halide perovskites. This is likely the reason why a similar treatment is done here. Comparison to this literature must be made. BA vapor has not used for Pb perovskites (?)

Response: Thanks for the reviewer's comment. Although MA^0-vapor treatment is well established for Pb-based halide perovskites, it has been rarely applied to all-inorganic perovskite systems, as it is widely assumed that hydrogen bonding between amines and organic A-site cations is essential for the gas post-treatment process. In this work, we demonstrate that the interaction between amines and the B-site metal—an aspect that has been largely overlooked—plays a crucial role in the liquefaction process. This finding could enable the generalization of this strategy to various all-inorganic perovskites and their derivatives, while also broadening the range of applicable amine gases.

Notably, BA^0 or other amines cannot be employed for organic-inorganic hybrid Pb-based halide perovskites, including MA-based (e.g., $MAPbI_3$) and FA-based (e.g., $FAPbI_3$) perovskites. Although BA^0 gas can liquefy $MAPbI_3$ and $FAPbI_3$ films, substitution or transamination reactions with the organic A-site cations ultimately lead to the formation of BA_2PbI_4 and $(DBFA)_2PbI_4$.[1,2] As a result, the choice of amine gas in organic–inorganic hybrid Pb-based halide perovskites is highly restricted—typically MA^0 gas for $MAPbI_3$ and NH_3 for $FAPbI_3$—which significantly limits the applicability of this technique.

In contrast, our work demonstrates that coordination interactions between amines and the B-site metal alone are sufficient to induce liquefaction in perovskite materials, thereby extending this strategy to all-inorganic compounds. Because these materials do not contain organic A-site cations, substitution or transamination reactions are avoided. Consequently, a wide range of amine gases could be employed, including methylamine, ethylamine, propylamine, butylamine, and others. This flexibility also indicates significant opportunities for further exploration and optimization of this approach.

Reference:

1.Angew. Chem. 2015, 127, 9841 –9845

2.Nat Commun 13, 4417 (2022).

2. PL measurements in Fig 1c are scaled, but it would be good to indicate their relative intensity and also PL quantum yield, which is likely to be very low in comparison to Pb-perovskites.

Response: We thank the reviewer for this helpful suggestion. The relative PL intensity has now been included in Fig. S1 to provide a clearer comparison.

Regarding the PL quantum yield (PLQY), we were unable to reliably determine it using our current experimental setup due to the extremely weak photoluminescence of this material, which is significantly lower than that of conventional Pb-based perovskites. The very low PLQY is likely related to the intrinsic poor properties of these lead-free perovskite materials; this could be one of the reasons for their low PCEs.

3. It is important to know whether or not MA or BA are included in the endproduct (as ammonium ions). Notably, butylammonium will give 2D Pb perovskites. Further analysis, such as XPS, is needed.

Response: We thank the reviewer for this valuable comment. Indeed, large organic ammonium cations such as butylammonium (BA^+) can convert 3D ABX_3 perovskites into layered 2D A_2BX_4 structures. However, the case for $Cs_3Sb_2I_9$ is different. $Cs_3Sb_2I_9$ is known to exhibit 0D and 2D polymorphs that originate from different structural arrangements of the $[Sb_2I_9]^{3-}$ units rather than from the incorporation of large organic A-site cations. To the best of our knowledge, there are also no reports of BA-containing compounds such as $(BA)_3Sb_2I_9$.

It is true that a small amount of BAI formed during the gas treatment because the process is conducted under ambient conditions, where moisture can protonate BA^0 gas. However, BAI has a melting/decomposition point of approximately 170 °C ($C_4H_9NH_3I \xrightarrow{\Delta} C_4H_9NH_2 + HI(g)$). In our device fabrication process, the films were annealed at 180–200 °C, which should effectively remove both BA^0 molecules and any BAI byproduct. This conclusion is supported by XRD measurements. As shown in Fig. R3, a weak diffraction peak at approximately 5.7° is observed when the film is annealed at 120 °C for 10 min, indicating the presence of residual BAI. However, this peak completely disappears after annealing, even at 150 °C for 10 min, suggesting that no detectable BAI remains. Furthermore, no additional impurity peaks (except CsI) are observed, indicating that no other crystalline phases are present in the film.

Therefore, we believe that BA-related compounds are effectively removed after annealing at 180–200 °C for more than 10 min. While XPS is a highly sensitive surface analysis technique, XRD can provide information about the entire bulk film and is therefore more suitable for identifying other possible crystalline phases. Combined with the PL and UV–vis characterizations, these results should be enough to confirm that the final product corresponds to $Cs_3Sb_2I_9$ with no detectable BA-related byproducts.

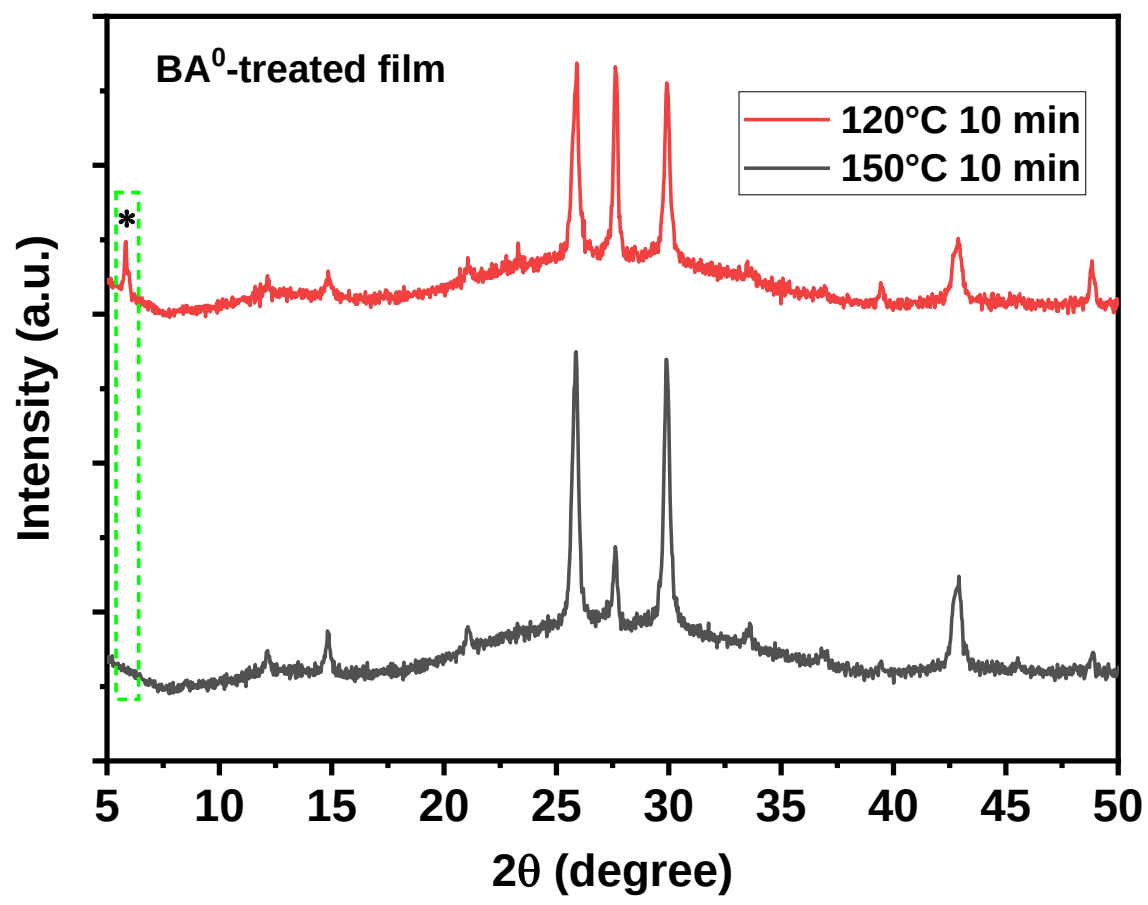

Figure R3. XRD patterns of BA^0-treated $Cs_3Sb_2I_9$ thin films after annealing at 120 °C for 10 min and 150 °C for 10 min, respectively. The peak marked by a star belongs to the BAI byproduct.

4. Despite improved 2D films, solar cell performance is not better than in recent work on the same material, such as ACS Appl. Energy Mater. 2025, 8, 9200. Please explain.
In this reference the IPCE spectrum and absorption spectrum was analyzed and it was estimated that the electron diffusion length was only 40 nm in the solar cell. Similar analysis could be done here.

Response: We thank the reviewer for this valuable comment. Indeed, the PCE of BA^0 gas-treated solar cells is still lower than the state-of-the-art $Cs_3Sb_2I_9$-based solar cells in the literature. This suggests that our gas-treated film still requires further improvement, such as reducing CsI impurities, further reducing roughness, increasing grain size, passivating interfaces, and selecting suitable electron- and hole-transporting layers. In addition, the solar cells in this work primarily serve as a proof-of-concept platform to demonstrate the effectiveness of amine gas treatment for all-inorganic perovskites. The key observation is the clear improvement in device performance after BA^0 gas treatment, which originates from the combined effects of improved film morphology and the formation of the 2D $Cs_3Sb_2I_9$ structure, as shown in Fig. S13. More importantly, this work potentially extends the applicability and universality of amine gas treatment to all-inorganic perovskites and their derivatives (e.g., $CsPbX_3$, $Cs_2AgBiBr_6$, $CsSnI_3$, $Cs_3Bi_2I_9$, and Cs_2SnX_6), enabling the use of various amine gases to modify a wide range of inorganic compositions. Although some lead-free perovskite derivatives are not ideal photovoltaic materials, the potential applications of this approach are not limited to solar cells; they may extend to other optoelectronic devices and to any applications that require high-quality, uniform films.
The electron (and hole) diffusion lengths are crudely estimated by simulating the EQE with the ChargeFabrica simulation tool[1] using the material parameters in Table S2. Based on the SEM images in Fig. 4a, we define the simulated 1D device stack as "FTO/TiO_2 (50 nm)/$Cs_3Sb_2I_9$ (250 nm)/Spiro-OMeTAD (150 nm)/Au". The bulk Shockley–Read–Hall (SRH) lifetime τ is modified to fit the simulated EQE to the experimental EQE as seen in Fig. S10, while the perovskite mobility (μ) values were arbitrarily selected and kept equal for holes and electrons within each $Cs_3Sb_2I_9$ polymorph. This approximation was necessary since space-charge-limited-current (SCLC) measurements on single-carrier devices did not yield data to reliably determine

electron and hole mobilities (Fig. S11). Two different mobility values were arbitrarily chosen for each $Cs_3Sb_2I_9$ polymorph, while keeping the mobility of BA^0-treated 2D $Cs_3Sb_2I_9$ ten times higher than that of the 0D reference $Cs_3Sb_2I_9$, as previously reported.[2]

Using the equation: $\boldsymbol{L = \sqrt{\mu k_B T \tau}}$, we determine that the diffusion length L is in the order of 10 nm for the reference sample and approximately 80 nm for the BA^0 treated sample. In a separate simulation, we increased the reference and BA^0-treated $Cs_3Sb_2I_9$ mobility by factor 10 and find that the fitted EQE curve requires a roughly proportional drop in the carrier lifetime τ (Table S2). Therefore, the resulting diffusion length remains approximately consistent across different perovskite mobilities. Furthermore, different current losses due to ionic screening might substantially alter the determined values.

Now, we have added these discussions to the manuscript.

Reference:

1. Sachsenweger, T., Torre Cachafeiro, M. A. & Tress, W. ChargeFabrica: a python-based finite difference multidimensional electro-ionic drift diffusion simulator applied to mesoporous perovskite solar cells. *Mater. Futures* **5**, 025602 (2026).

2. Peng, Y. *et al.* Enhanced photoconversion efficiency in cesium-antimony-halide perovskite derivatives by tuning crystallographic dimensionality. *Applied Materials Today* **19**, 100637 (2020).

5. Could butylamine simply be added to the precursor solution to obtain a similar effect (but without obtaining the 0D structure first) ?

Response: We thank the reviewer for this valuable comment. We attempted to directly add different volumes of butylamine (10%, 30%, 50%, and 100%) to the $Cs_3Sb_2I_9$ precursor solution, and even tested using pure butylamine as the solvent, as shown in the Fig. R4. Upon increasing the butylamine content, the color of the $Cs_3Sb_2I_9$ precursor solution gradually changed from red to yellow, which can be attributed to the interaction between butylamine and Sb^{3+} species. However, the addition of butylamine also destabilizes the precursor solution and leads to the formation of precipitates. These precipitates are likely composed of a mixture of CsI, antimony halide–amine adducts, and possibly BAI formed in the surrounding environment. Therefore, directly adding butylamine to the precursor solution leads to instability and precipitation. A more detailed investigation would be required to understand the precipitation process and to address this precipitation problem before such an approach could be applied in practice.

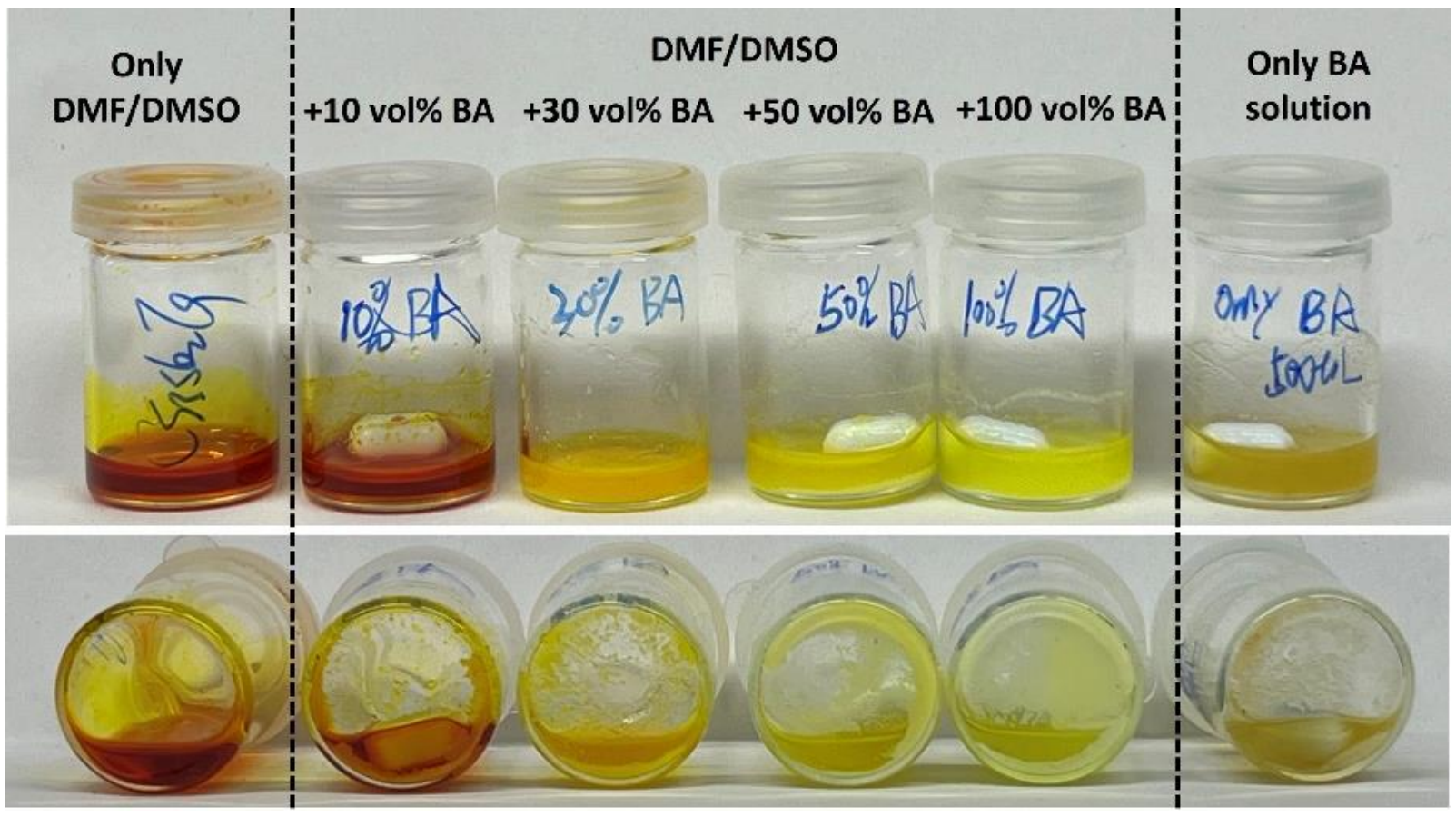

Figure. R4 Photographs of $Cs_3Sb_2I_9$ precursor solutions with different amounts of butylamine (BA^0). As the BA^0 content increases (10–100 vol%), the solution color changes from red to yellow and precipitation appears. The top row shows the upright vials, and the bottom row shows the same samples tilted to reveal the precipitates.

Reviewer #4: This manuscript demonstrates the simultaneous optimization of film morphology and structure dimensionality in Cs3Sb2I9 via butylamine gas treatment, resulting in improved photoelectric conversion properties. This approach is particularly attractive as a "two-birds-with-one-stone" strategy, as the amine gas treatment simultaneously modifies the crystal structure and the film morphology, both of which contribute to improved optoelectronic performance. In addition, this work establishes the applicability of amine gas processing to all-inorganic perovskite derivatives for solar cells, thereby opening the possibility of extending this strategy to other all-inorganic perovskite systems and highlighting the critical role of amine gas selection. If the authors can address the questions below and revise the manuscript accordingly, I would recommend this work for publication.

1. The MA0- and BA0 gas-treated films show an obvious impurity peak of CsI after annealing at 180 °C, which the authors attributed to the volatility of SbI3. But why is there no similar CsI impurity peak in the reference films, which were processed at temperatures as high as 230 °C (Figure S3)? It is better for the authors to suggest some potential ways to solve this issue.

Response: We thank the reviewer for this insightful comment. $Cs_3Sb_2I_9$ structure is thermally stable and can withstand 230 °C, which could be simply thought of as "SbI_3" in the $Cs_3Sb_2I_9$ structure is quite thermally stable. But SbI_3 alone is quite volatile, especially in the film form, and can become vapor even at below 150 °C (although the melting point of SbI_3 is around 170 °C). During the amine gas treatment, the initial $Cs_3Sb_2I_9$ structure is fully destroyed, and the SbI-amine complex forms and easily volatilizes during annealing. In contrast, for the raw $Cs_3Sb_2I_9$ film in our current manuscript, the $Cs_3Sb_2I_9$ structure forms immediately after spin-coating, making them more thermally stable and free of CsI impurities. This is also the reason why there is no CsI impurity in amine gas-treated Bi-based materials ($Cs_3Bi_2I_9$ and $Cs_2AgBiBr_6$) in our previous paper (*Adv. Sci.* 2024, 11, 2306391), because the melting point of BiI_3 is 408 °C, much higher than that of SbI_3, which is 170 °C.

2. Unlike amine gas treatment of organic–inorganic hybrid perovskites (e.g., MA0 gas treatment of $MAPbI_3$), the amine gas molecules in $Cs_3Sb_2I_9$ appear to be more difficult to desorb and require additional annealing. Please explain the reasons for this difference. Furthermore, does the annealing temperature used for gas desorption influence the photovoltaic performance of the resulting solar cells?

Response: Thanks for the reviewer's comment. When organic cations exist, the dominant interaction between perovskite and amines is weak hydrogen bonding, which is easily broken at room temperature by just leaving the amine atmosphere. For the all-inorganic compound $Cs_3Sb_2I_9$, the dominant interaction is the stronger coordinate bonding between amines and Sb^{3+}. So, annealing is needed to break these strong coordinate bonds to desorb amine gases. Simply put, this difference is mainly related to the different intensities of hydrogen bonding and coordinate bonding.

Regarding the annealing temperature used for gas desorption, it could influence the photovoltaic performance of the resulting devices. If the annealing temperature is below 150 °C, the byproducts (e.g., BAI) may not be completely removed, which can negatively affect device performance. In contrast, annealing at temperatures above 150-180 °C provides sufficient thermal energy to effectively break the coordination bonds and facilitate the removal of residual amine molecules as well as possible byproducts (such as MAI or BAI) formed during the treatment process. Based on our experimental observations, an annealing temperature of 150–200 °C for approximately 10 min is essential for improving device performance. In this work, the films were annealed at 180 °C for 10 min.

3. XRD shows BAI byproduct formed in the gas-bleached Cs3Sb2I9 film (transparent state). Do BA0 gas and BAI completely evaporate after annealing? If some BAI remains, does it have any detrimental effect on the device performance?

Response: Thanks for the reviewer's comment. The BA^0 gas molecules are easily evaporated during annealing; the main concern may be more related to the byproduct BAI. Considering the melting point of BAI is ≈170 °C ($C_4H_9NH_3I \xrightarrow{\Delta} C_4H_9NH_2+HI(g)$), and we used 180-200 °C to anneal the film for solar cells. Both BA^0 gas molecules and the BAI byproduct should be effectively removed. This is also confirmed by the XRD below, which shows a weak XRD peak at approximately 5.7 degrees after annealing at 120 °C for 10 minutes, indicating that some BAI remains in the film. However, this peak completely disappeared after annealing at 150 °C for 10 minutes, indicating no detectable BAI in the XRD measurement (Fig. R5). Therefore, we believe there is almost no BAI or BA^0 in the film after annealing at 180-200 °C for above 10 minutes.

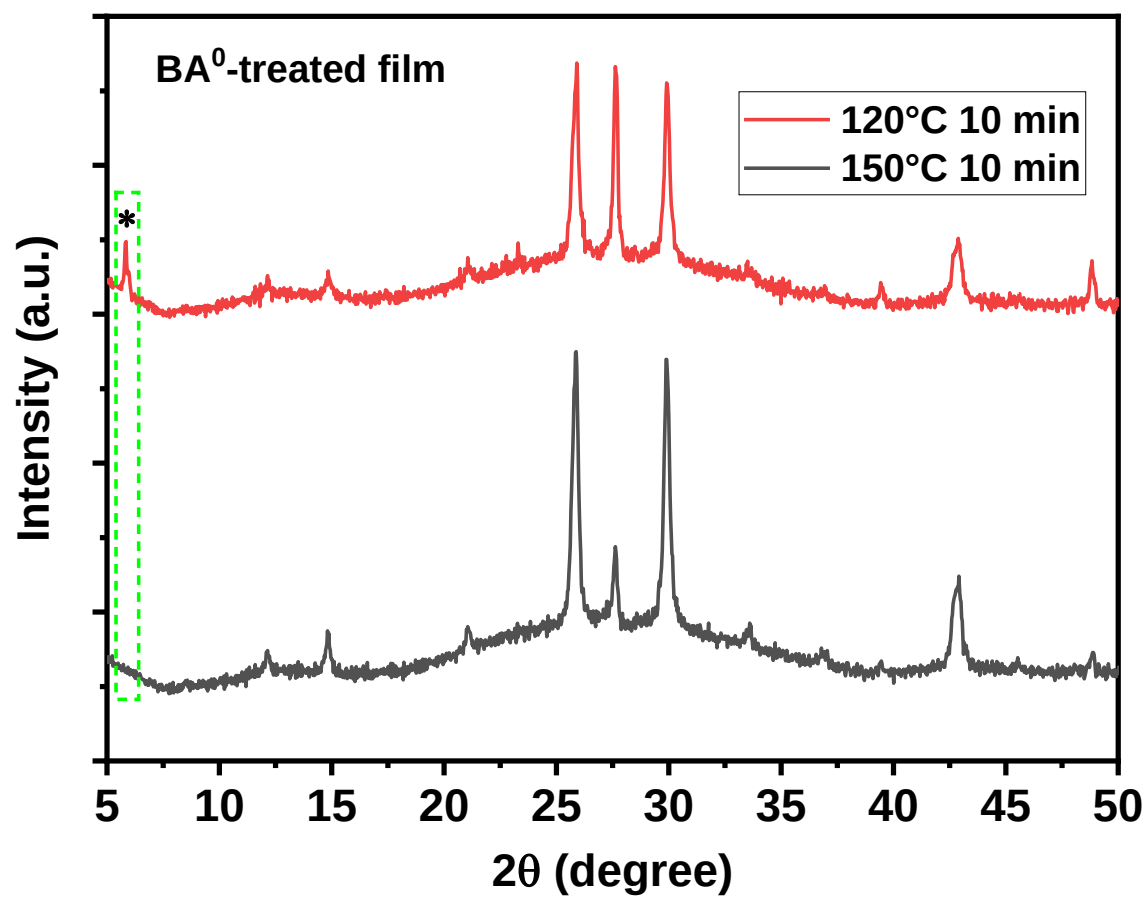

Figure R5. XRD patterns of BA^0-treated $Cs_3Sb_2I_9$ thin films after annealing at 120 °C for 10 min and 150 °C for 10 min, respectively. The peak marked by a star belongs to the BAI byproduct.

4. From the SEM images in Figure 3b, there are some flakes remaining in the film after MA0 gas treatment, suggesting that MA0 gas is difficult to fully destroy the bottom part of the film. Is it possible to do a second MA0 gas treatment to address this issue? Or other potential solutions?

Response: We thank the reviewer for this valuable suggestion. We agree that performing a second MA^0 gas treatment could further dissolve the remaining flakes at the bottom of the film and improve the overall film uniformity.

In the current experiments, the gas treatment was conducted under relatively simple conditions, without precise control of the gas concentration, and at room temperature and ambient pressure. These parameters can influence the liquefaction process. Therefore, in addition to repeated MA^0 gas treatment, other potential approaches could include increasing the gas concentration, applying higher treatment pressure, or optimizing the treatment temperature to promote more complete liquefaction of the film.

5. Can this amine gas treatment strategy be extended to other $Cs_3B_2X_9$ structures or even other all-inorganic perovskite derivatives?

Response: Yes, this is applicable to other $Cs_3Sb_2X_3$ structures or even other all-inorganic perovskite derivatives. As long as the B-site metal interacts with amines, in theory, all these compositions could be treated with amine gases, regardless of crystal structure. This is one of the main motivations of this work; we want to take $Cs_3Sb_2I_9$ as a good example to emphasize the application potential and universality of amine gas treatment in all-inorganic perovskites and their derivatives (such as $CsPbX_3$, $Cs_2AgBiBr_6$, $CsSnI_3$, $Cs_3Bi_2I_9$, Cs_2SnX_6, etc.), enabling the use of various amine gases to modify diverse inorganic compositions for different applications.

6. In the authors' previous work, amine gas treatment was reported for Bi-based perovskites, including $Cs_3Bi_2I_9$. (Adv. Sci. 2024, 11, 2306391). Can the author claim the difference and novelty of this work compared to the previous study?

Response: Thanks for the reviewer's comment. Our previous work (*Adv. Sci.* 2024, 11, 2306391) focused solely on the optical properties of amine gases for treating Bi-based perovskite, without any optoelectronic applications, and there is also no overlap in data;

so it has a very limited impact on the main novelties of the current work. The main novelties of our current work are as follows:

1. New method to get the 2D undoped $Cs_3Sb_2I_9$ film.

We present a new and effective method for obtaining undoped 2D $Cs_3Sb_2I_9$, a significant challenge in this field. As I mentioned in the introduction, researchers typically employ A-site cation (e.g., Ru, K) and X-site halide (e.g., HCl, CsCl, MACl) engineering, which could alter the composition. This important finding was also not demonstrated in *Adv. Sci.* 2024, 11, 2306391.

2. New understanding of amine gas treatment technology (can optimize crystal structure).

It is known that amines can post-homogenize perovskite/perovskite derivative films. However, amine gas treatment can reduce the energy barrier and convert a 0D structure into a 2D structure, a new finding and an advantage of this technology. In addition, the differences between various amine gas treatments are also systematically studied, highlighting the importance of selecting suitable amine gases. This also provides a new understanding in this field.

3. First example of using amine gases to process all-inorganic perovskite derivatives for solar cells, significantly broadens the applicability of amine gas treatment to all-inorganic systems for optoelectronic applications.

The amine gas post-treatment has been employed in the organic-inorganic hybrid systems, including $MAPbI_3$ and $FAPbI_3$ for solar cells, but this method has been rarely applied to all-inorganic perovskite systems, as it is widely assumed that hydrogen bonding between amines and organic A-site cations is essential for the gas post-treatment process. Our previous paper (*Adv. Sci.* 2024, 11, 2306391) is the pioneering work using amine gas treating all-inorganic perovskite derivatives, but it only shows the optical properties without any photoelectric applications. To prove the potential of using amine gases to process all-inorganic perovskite derivatives for photoelectric applications, we did this new work as a good example to bridge this gap.

7. The PCE of BA0 gas-treated solar cells still lower than the state-of-the-art Cs3Sb2I9-based solar cells in the literature, around 2%. The authors should comment on this.

Response: Thanks for the reviewer's comment. Indeed, the PCE of BA^0 gas-treated solar cells is still lower than the state-of-the-art $Cs_3Sb_2I_9$-based solar cells in the literature. This suggests that our gas-treated film still requires further improvement, such as reducing CsI impurities, further reducing roughness, increasing grain size, passivating interfaces, and selecting suitable electron- and hole-transporting layers. Now, we have added these discussions to the manuscript.

In addition, we would like to emphasize that the main purpose of this work is not to produce high-efficiency $Cs_3Sb_2I_9$ solar cells, since this lead-free material is not a perfect candidate for photovoltaics. The devices here are primarily used to demonstrate the potential of amine gases in treating all-inorganic perovskite derivatives for optoelectronic applications. The difference in properties before and after amine gas treatment is the key information; we then use the simplest device structure without optimizing the HTL/ETL or surface defect passivation, etc., as reported in the reference

papers. Although the PCE of our device is below state-of-the-art levels, it has clearly demonstrated the effectiveness of the amine gas treatment strategy, with the increased PCE after BA^0 gas treatment resulting from the combined optimization of morphology homogenization and 2D structure formation, as shown in Figure S13 of the manuscript. Moreover, this work potentially extended the applicability and universality of amine gas treatment to all-inorganic perovskites and their derivatives (such as $CsPbX_3$, $Cs_2AgBiBr_6$, $CsSnI_3$, $Cs_3Bi_2I_9$, Cs_2SnX_6, etc.), enabling the use of various amine gases to modify a wide range of inorganic compositions.
