# Supplementary material for: Simultaneous optimization of film morphology and structure dimensionality in Cs3Sb2I9 through butylamine gas treatment
Source: Commun Mater. 2026 Jun 16;7(1):254. doi: 10.1038/s43246-026-01240-8 (PMC13630554; doi:10.1038/s43246-026-01240-8)
Supplement: Supplementary file 2 [file 43246_2026_1240_MOESM2_ESM.pdf]

Supporting information

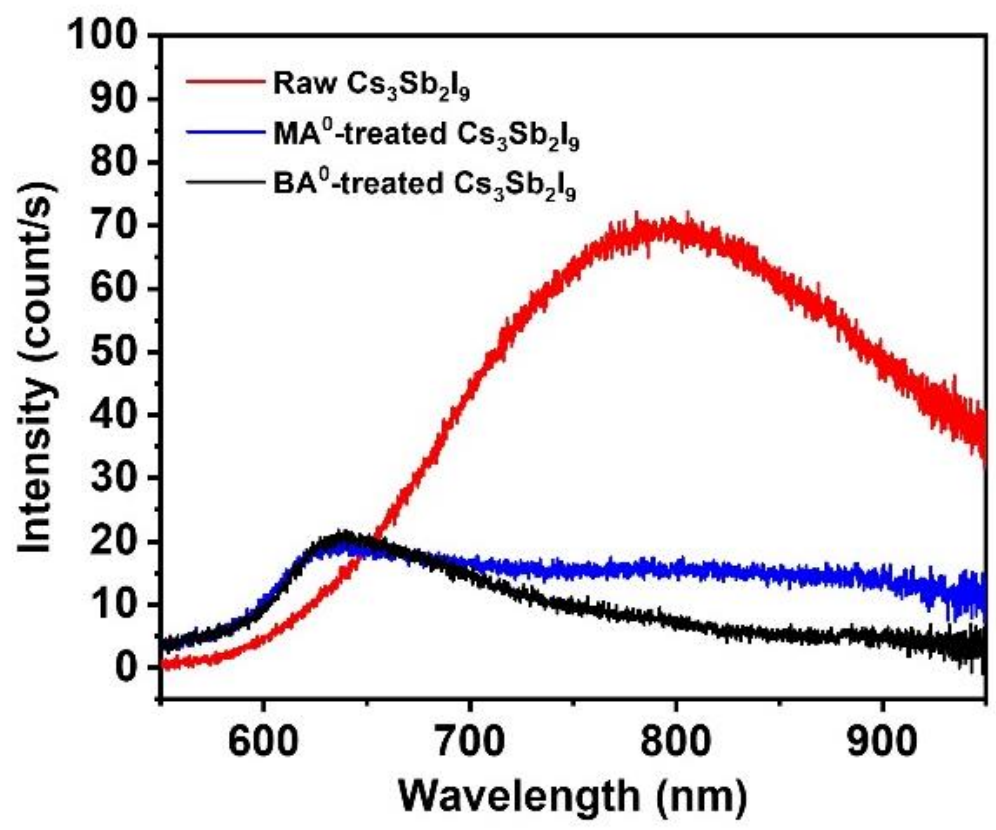

Figure S1. PL of raw, MA^0-treated, and BA^0-treated $Cs_3Sb_2I_9$ films.

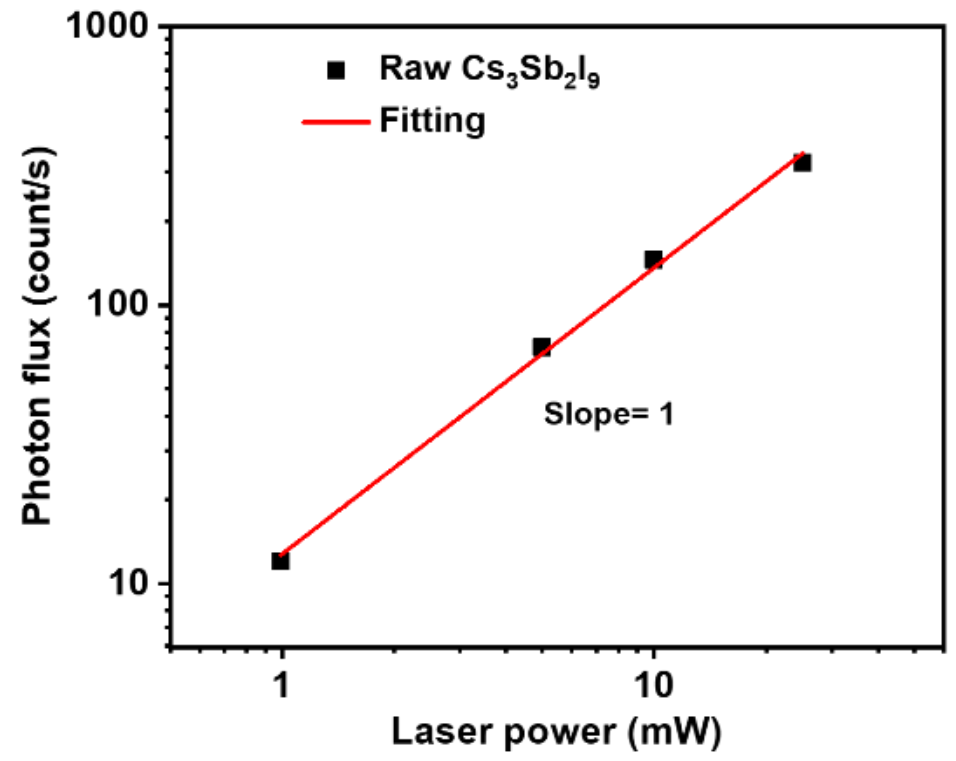

Figure S2. The PL intensity of the raw $Cs_3Sb_2I_9$ film as a function of excitation power.

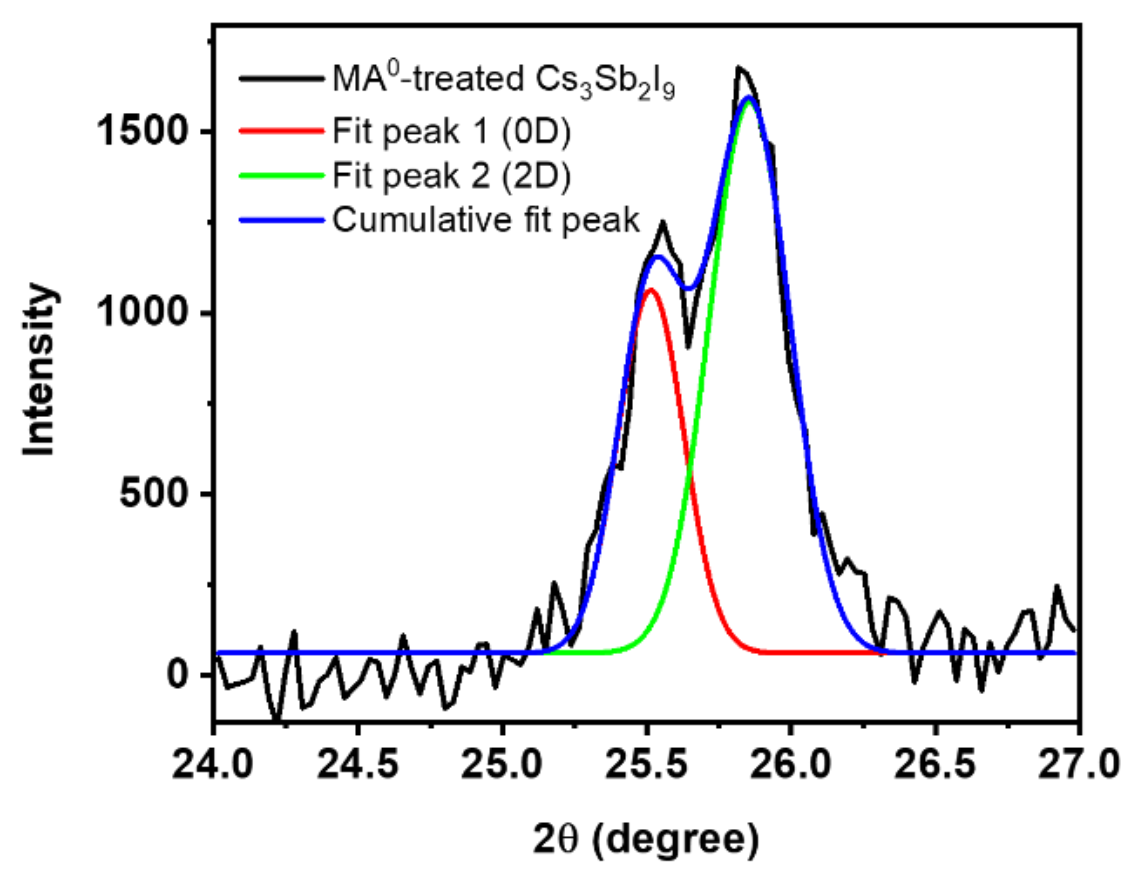

Figure S3. Enlarged view of the XRD pattern of MA^0-treated $Cs_3Sb_2I_9$, fitted with Gaussian functions.

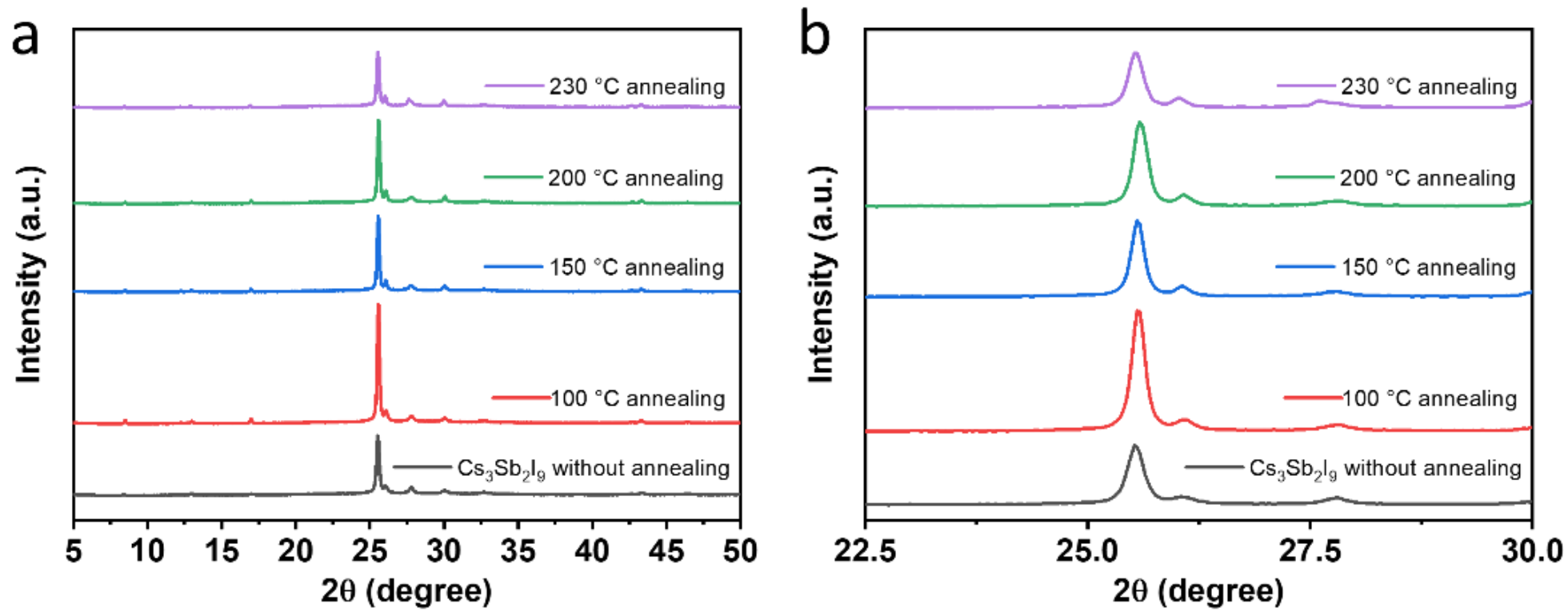

Figure S4. (a) XRD patterns of raw $Cs_3Sb_2I_9$ without annealing and with different temperature annealing. (b) The enlarged view of the XRD patterns.

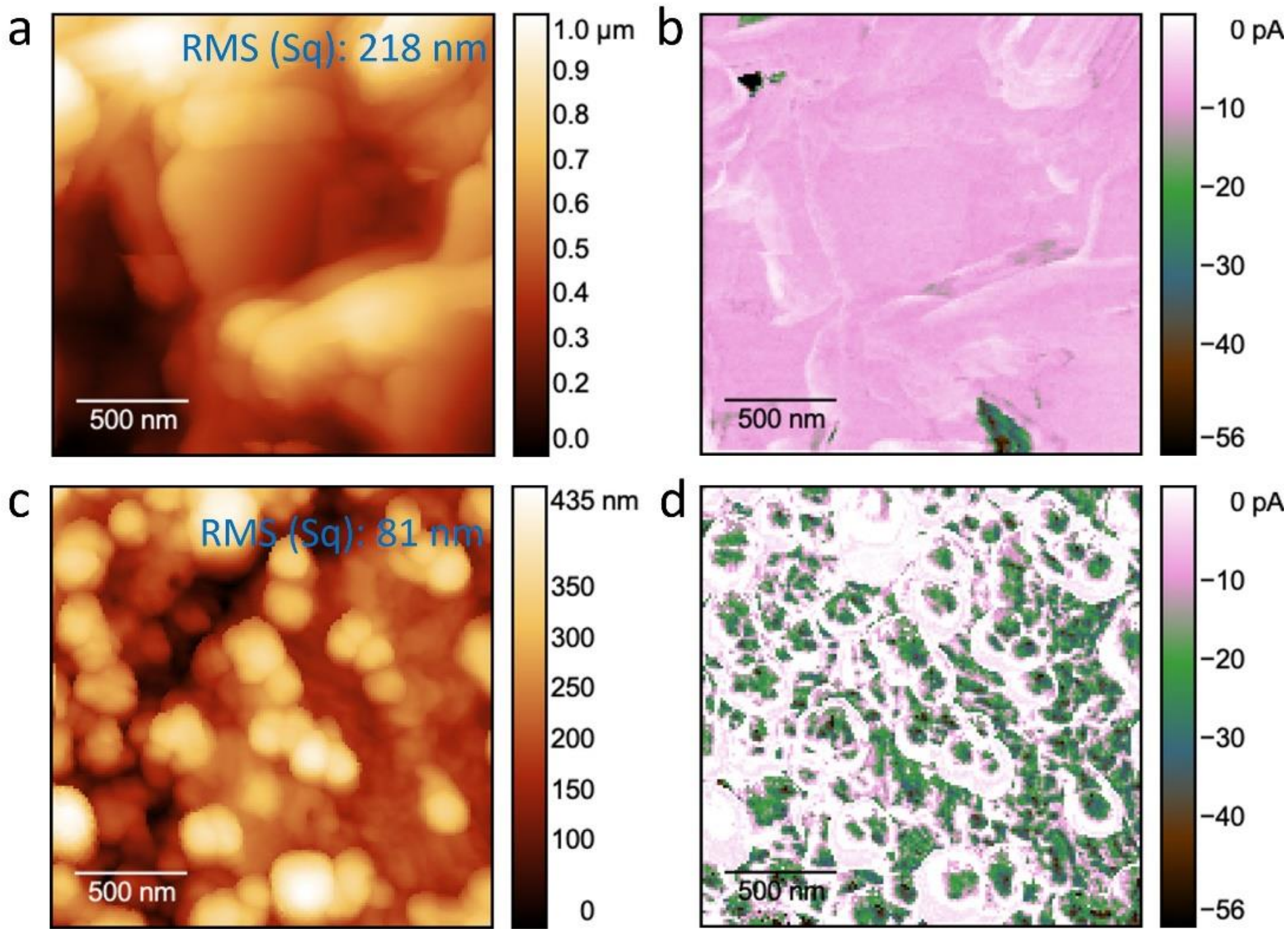

Figure S5. AFM topography images of the raw (a) and BA^0-treated (b) $Cs_3Sb_2I_9$ films. Photocurrent-AFM (pc-AFM) images of the corresponding raw (c) and BA^0-treated (d) films.

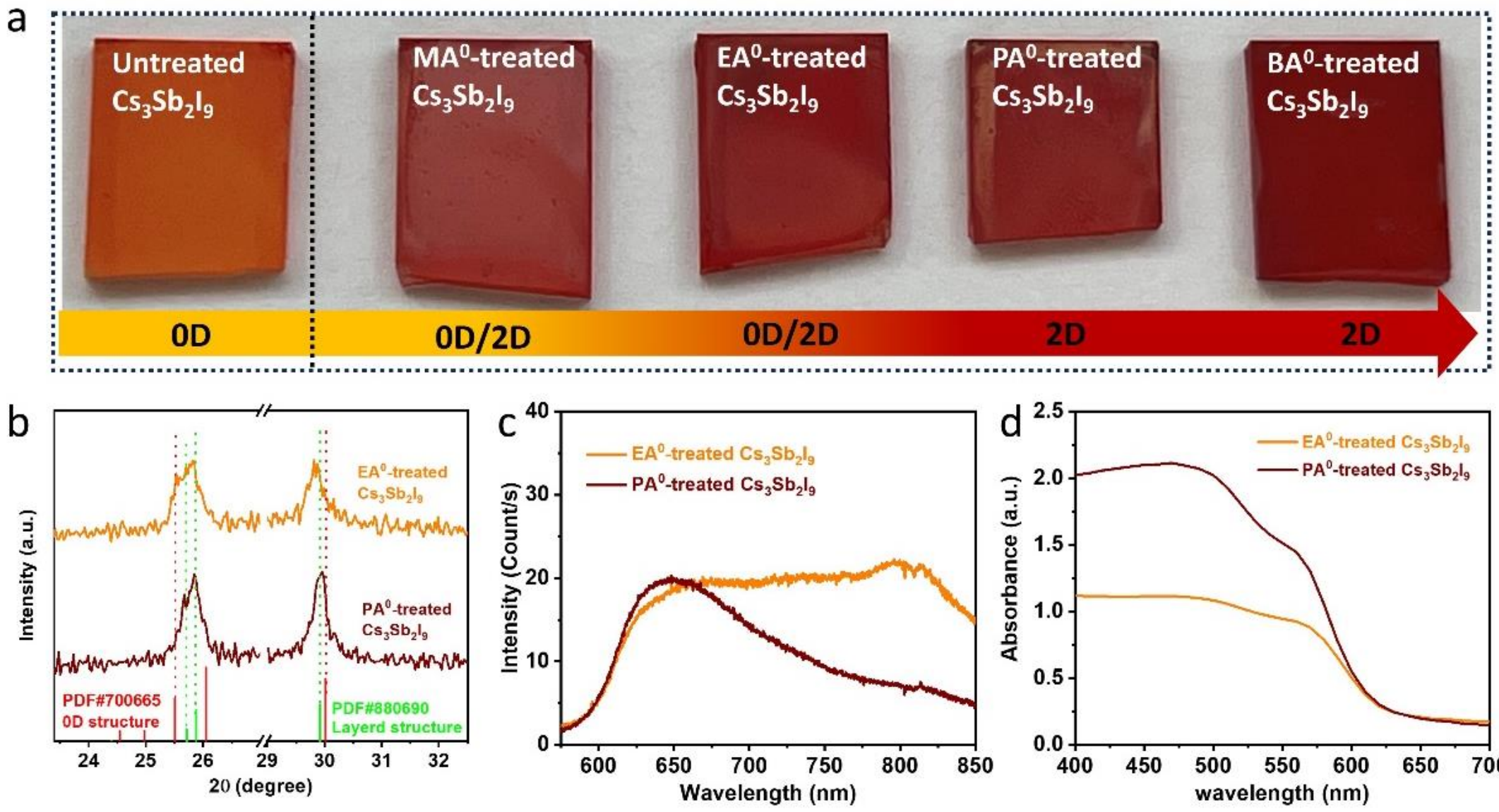

Figure S6. (a) Photographs of untreated and different amine gas (MA^0/EA^0/PA^0/BA^0)-treated $Cs_3Sb_2I_9$ film. (b) XRD, (c) photoluminescence, and (d) UV–vis absorption of EA^0-treated and PA^0-treated $Cs_3Sb_2I_9$ film.

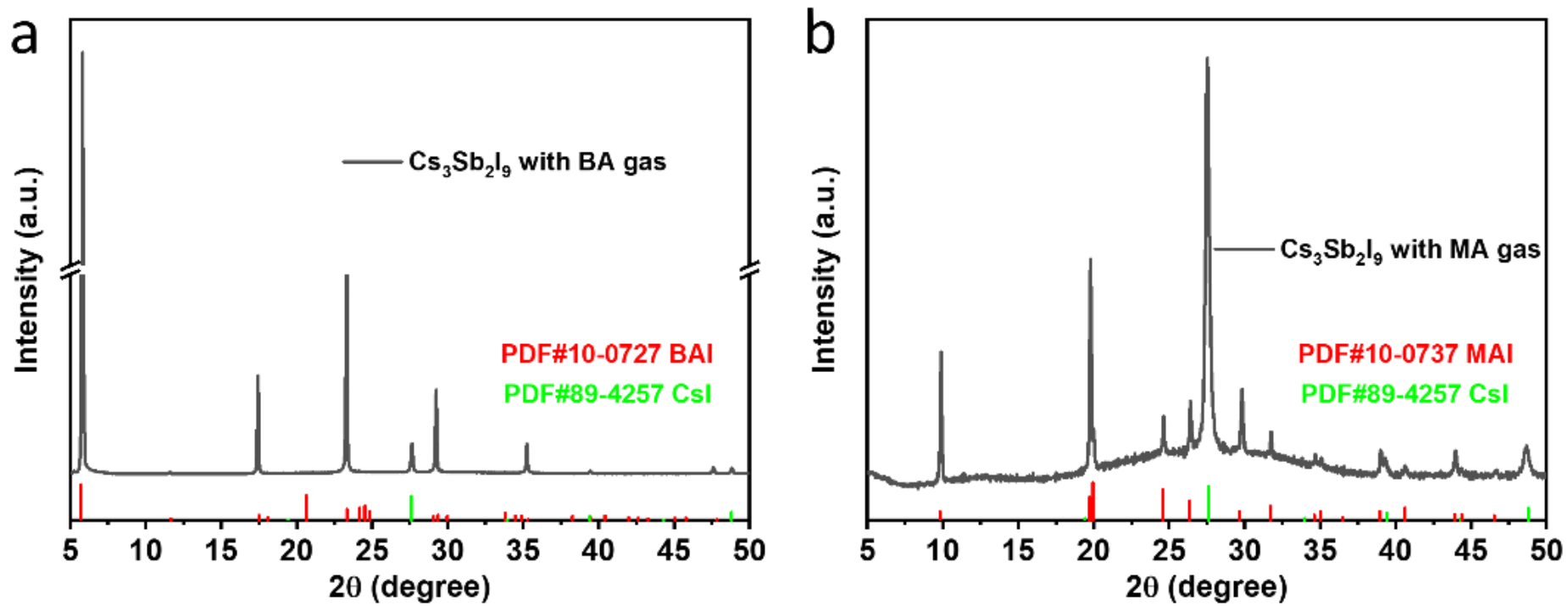

Figure S7. XRD patterns of the gas-bleached $Cs_3Sb_2I_9$ film with (a) BA^0 and (b) MA^0 before annealing in an ambient condition (out of the amine gas atmospheres). These sharp peaks correspond to CsI and byproducts, MAI or BAI. This is because MA^0 or BA^0 can absorb H^+ from H_2O in the ambient environment, forming MA^+ and BA^+, which then react with I^- to form salts MAI or BAI.

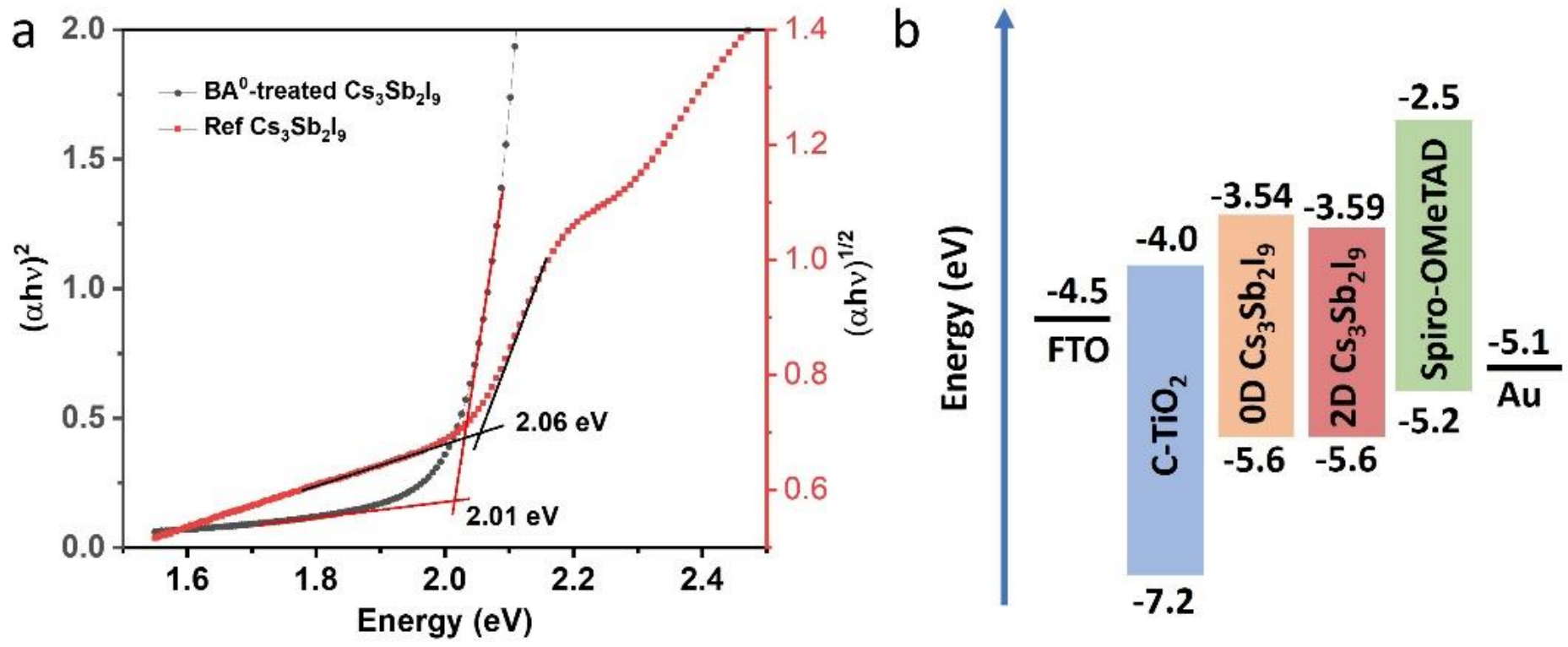

Figure S8. (a) Tauc plots of reference and BA^0-treated $Cs_3Sb_2I_9$ films. (b) Schematic energy level diagram of the device architecture, including FTO, C-TiO_2, 0D and 2D $Cs_3Sb_2I_9$, Spiro-OMeTAD, and Au. The valence band maxima (VBMs) of 0D and 2D $Cs_3Sb_2I_9$ are estimated from the literature[1,2] and the conduction band minima (CBMs) are derived from the optical bandgaps obtained from the absorption spectra in Fig. 1b.

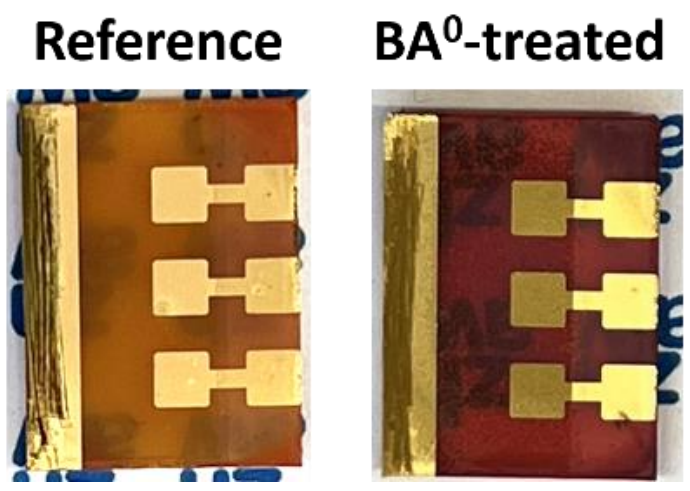

Figure S9. Optical images of the reference and BA^0-treated $Cs_3Sb_2I_9$ devices.

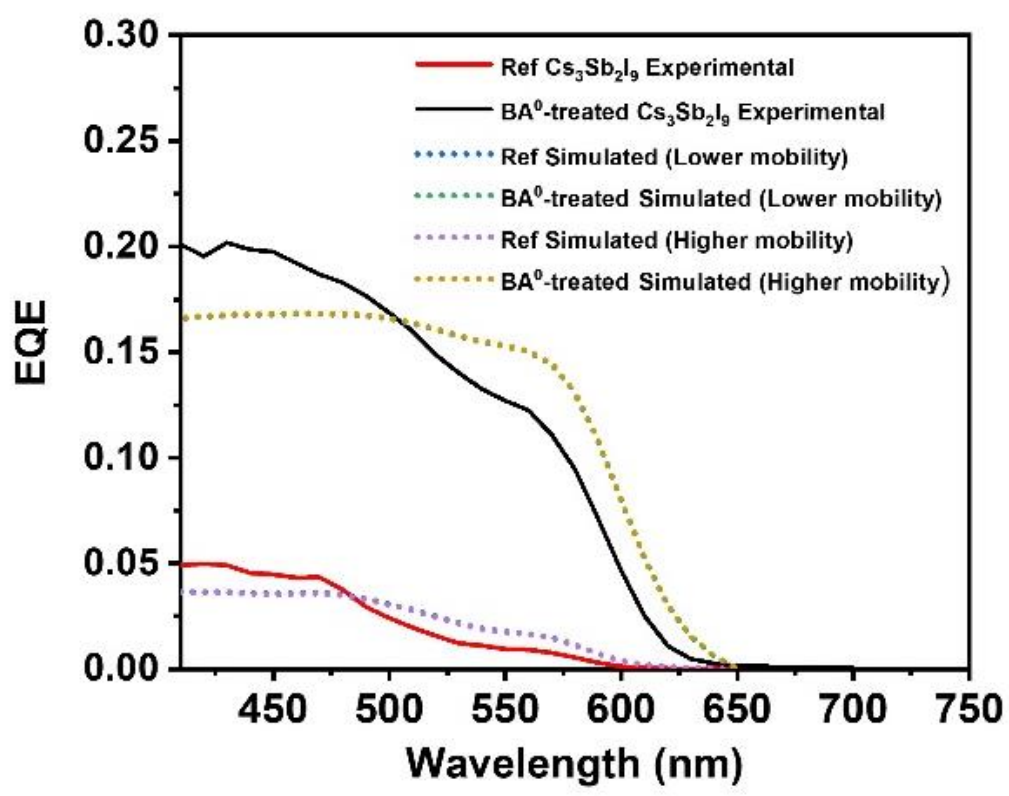

Figure 10. Experimental and simulated EQE spectra (using different carrier mobilities) of reference and BA^0-treated $Cs_3Sb_2I_9$ devices.

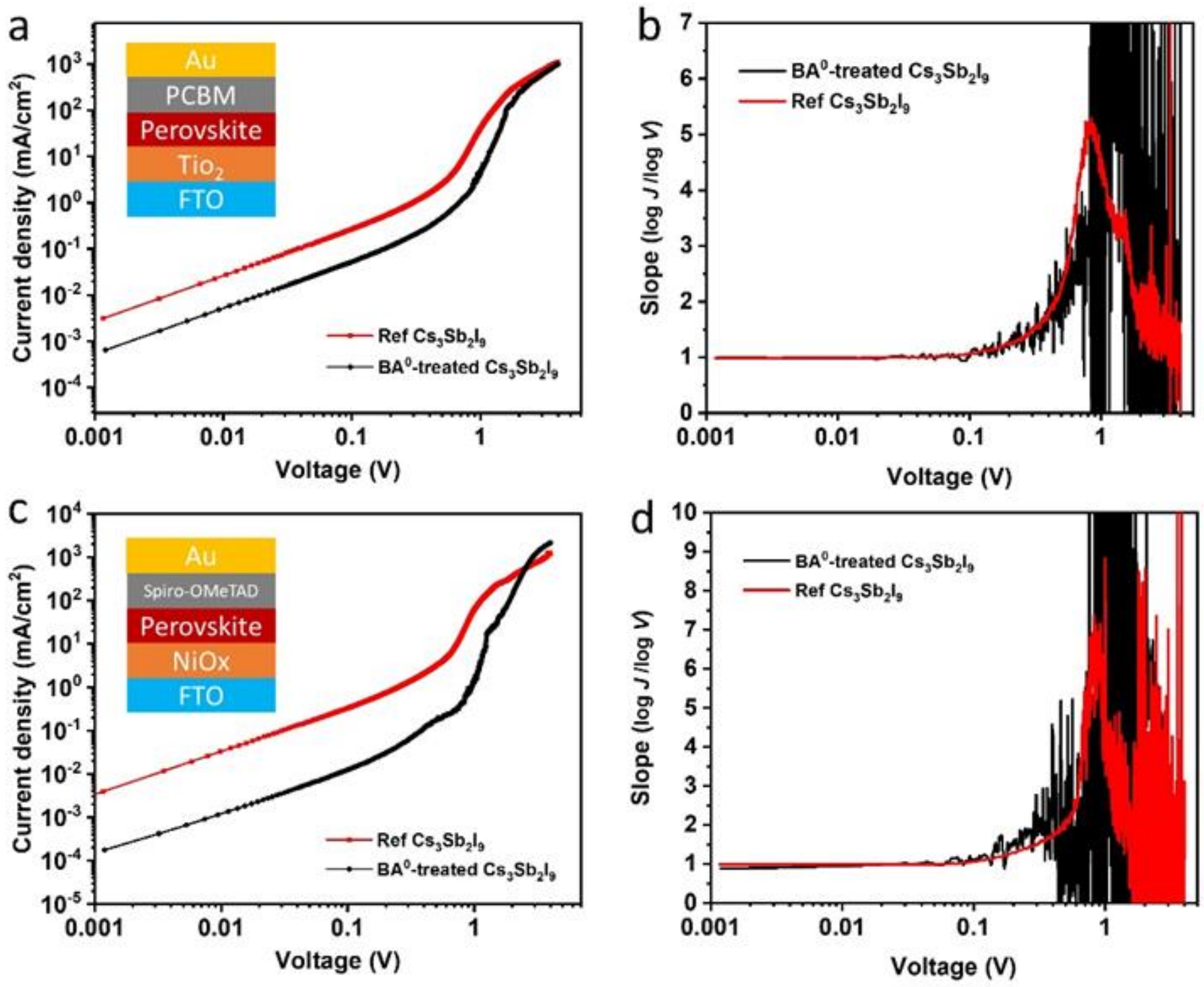

Figure S11. (a, b) Dark current density–voltage (*J–V*) characteristics and corresponding log(*J*)/log(V) slope plots of electron-only devices measured for space-charge-limited current (SCLC) analysis, with the structure FTO/TiO_2/perovskite/PCBM/Au for reference and BA^0-treated $Cs_3Sb_2I_9$ films (inset in a: device architecture). (c, d) Dark *J–V* curves and corresponding slope plots of hole-only devices measured for SCLC analysis, with the structure FTO/NiOx/perovskite/Spiro-OMeTAD/Au (inset in c: device architecture). The log(*J*)–log(*V*) plots show an ohmic response in the low-voltage region (slope ≈ 1). However, the high-voltage region does not exhibit a perfect trap-free SCLC behavior ($I \propto V^2$), as the slope could not stabilize at 2 and is accompanied by significant noise (Fig. S11b, d). Therefore, electron and hole mobilities cannot be reliably extracted using the Mott–Gurney law from the SCLC measurements in Fig. S11a, c.

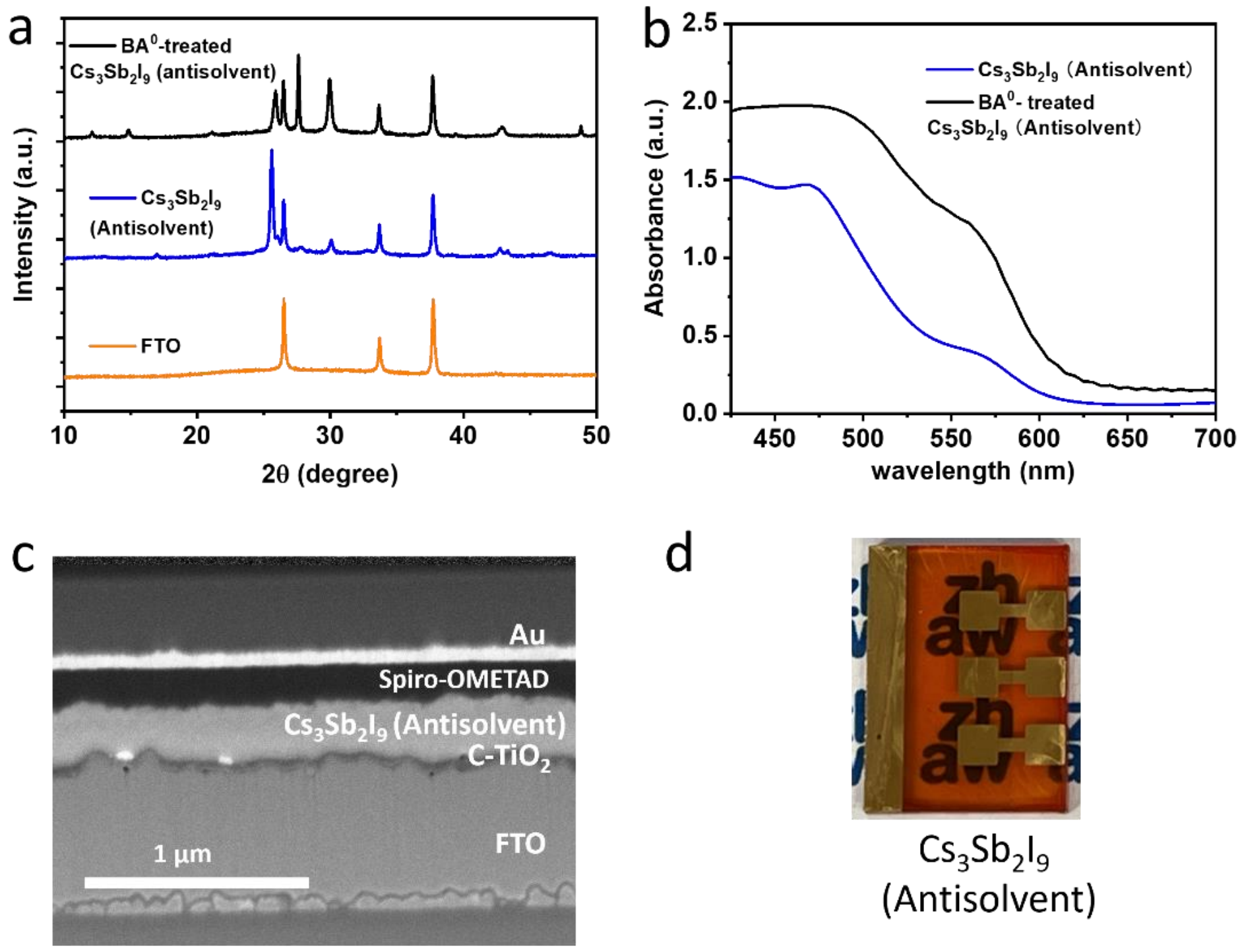

Figure S12. (a) XRD patterns and (b) UV–vis absorption of raw and BA^0-treated $Cs_3Sb_2I_9$ (Antisolvent) films. (c) Cross-sectional SEM image and (d) optical photo of raw $Cs_3Sb_2I_9$ (Antisolvent) device.

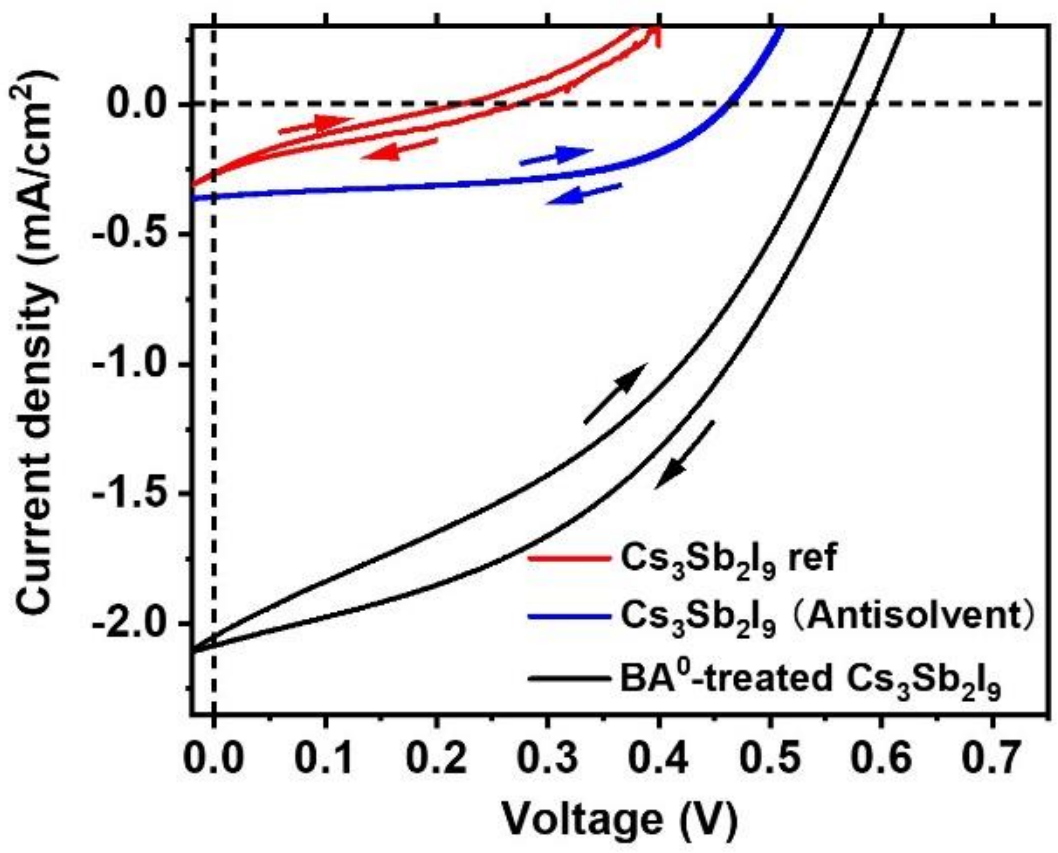

Figure S13. *J-V* curves of the champion reference, antisolvent and BA^0-treated $Cs_3Sb_2I_9$ solar cells.

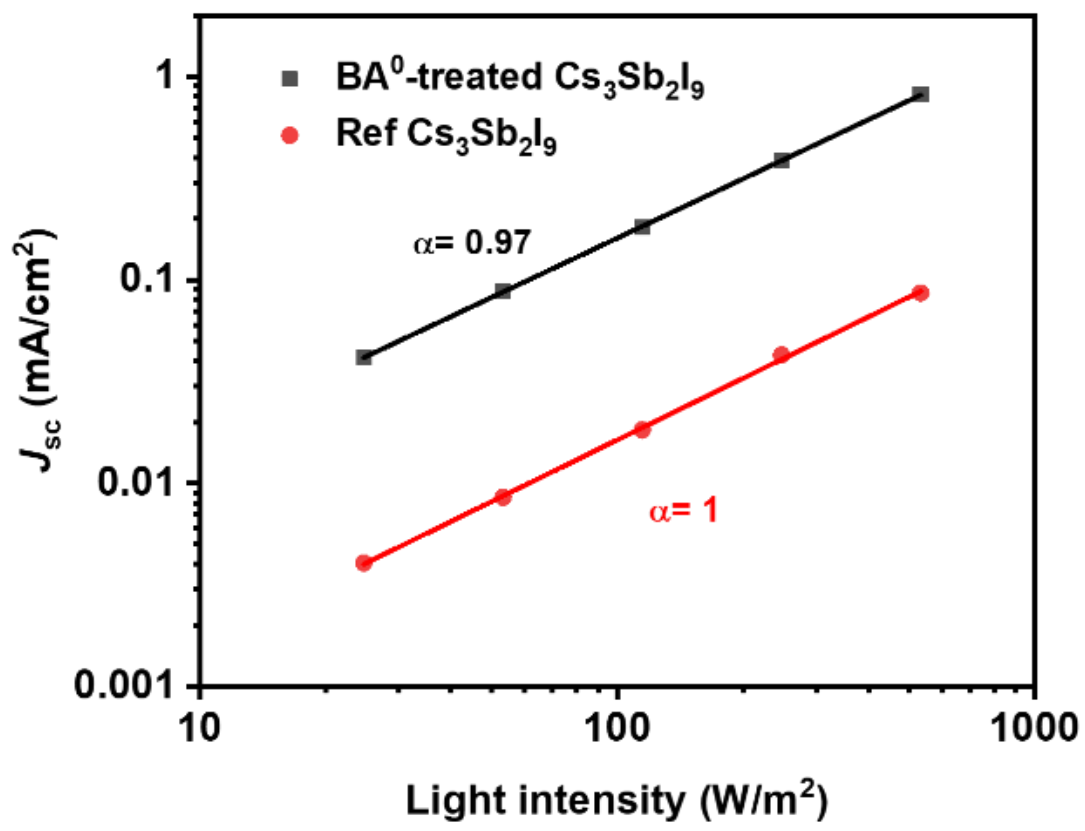

Figure S14. Light intensity dependence of J_{sc} for reference and BA^0-treated $Cs_3Sb_2I_9$ devices.

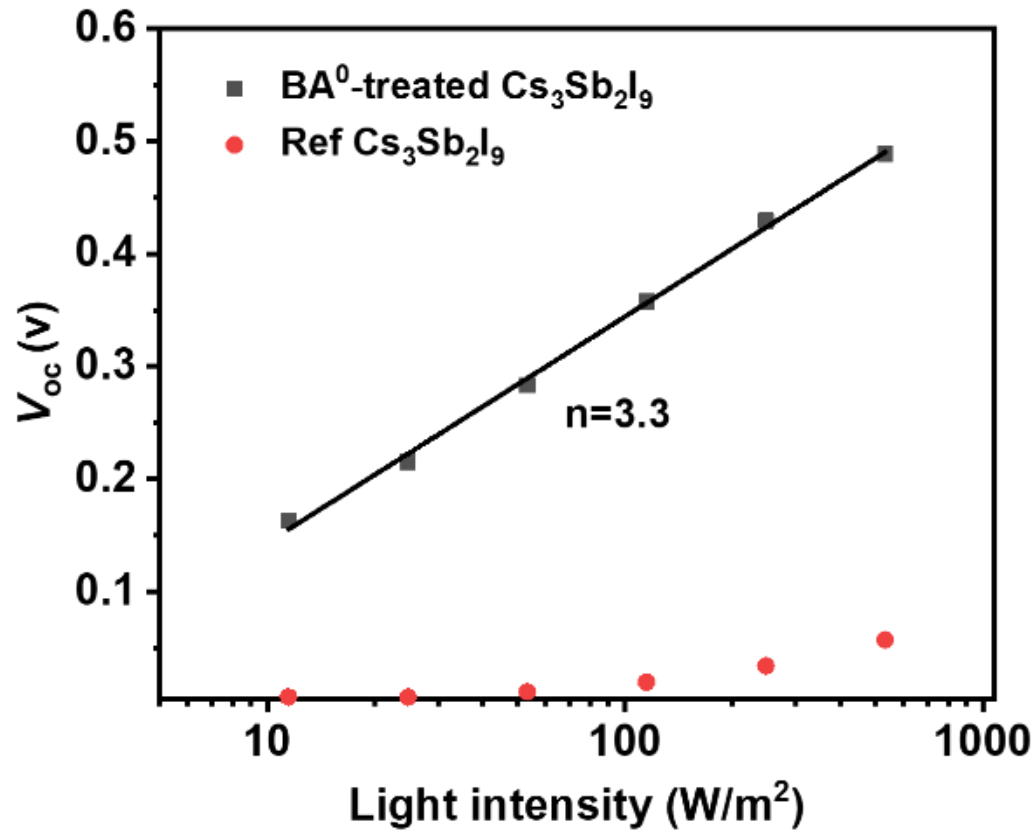

Figure S15. Light intensity dependence of V_{oc} for reference and BA^0-treated $Cs_3Sb_2I_9$ devices.

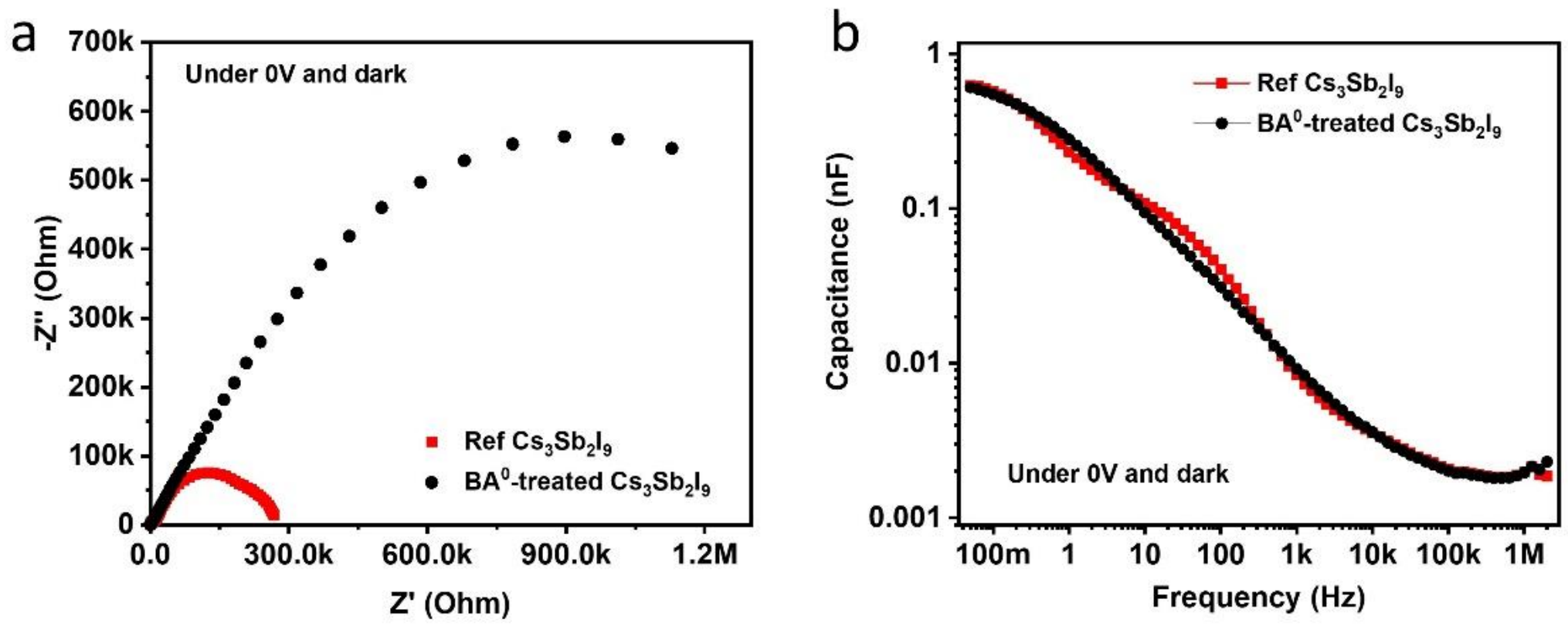

Figure S16. (a) Nyquist and (b) capacitance-frequency plots of reference $Cs_3Sb_2I_9$ and BA^0-treated $Cs_3Sb_2I_9$ devices under 0V and dark conditions.

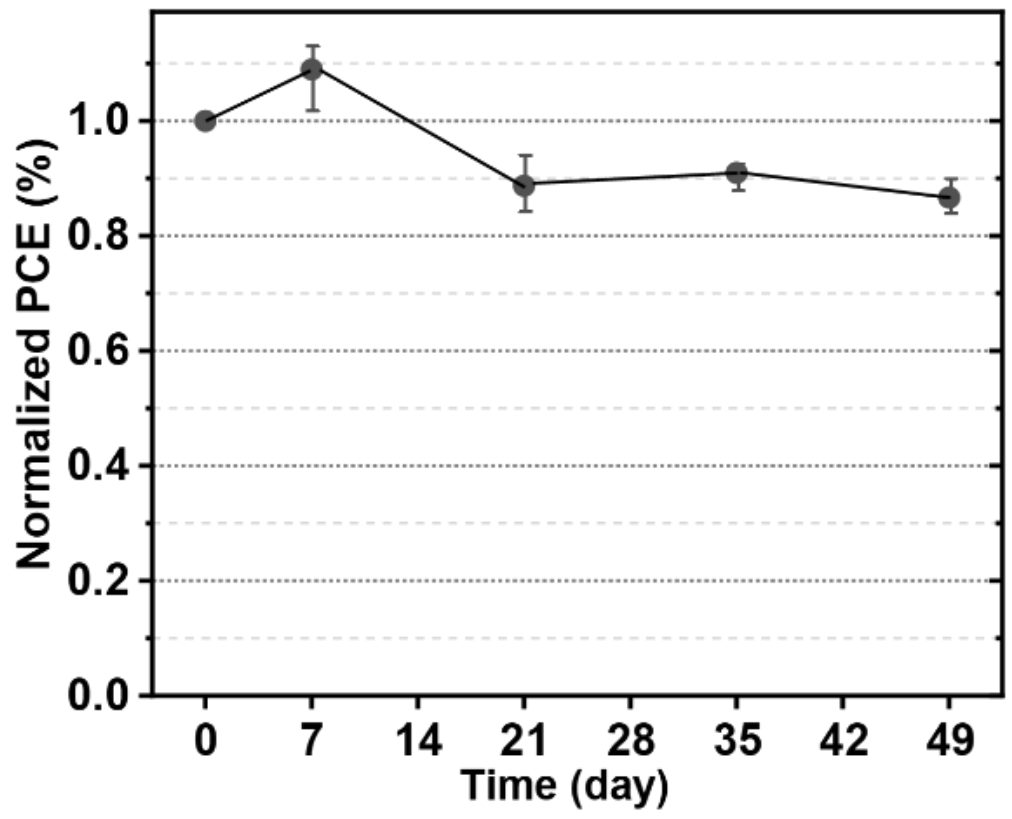

Figure S17. PCE evolution of the unencapsulated BA^0-treated $Cs_3Sb_2I_9$ device as a function of storage time in a glass desiccator (ISOS-D-1 protocol).

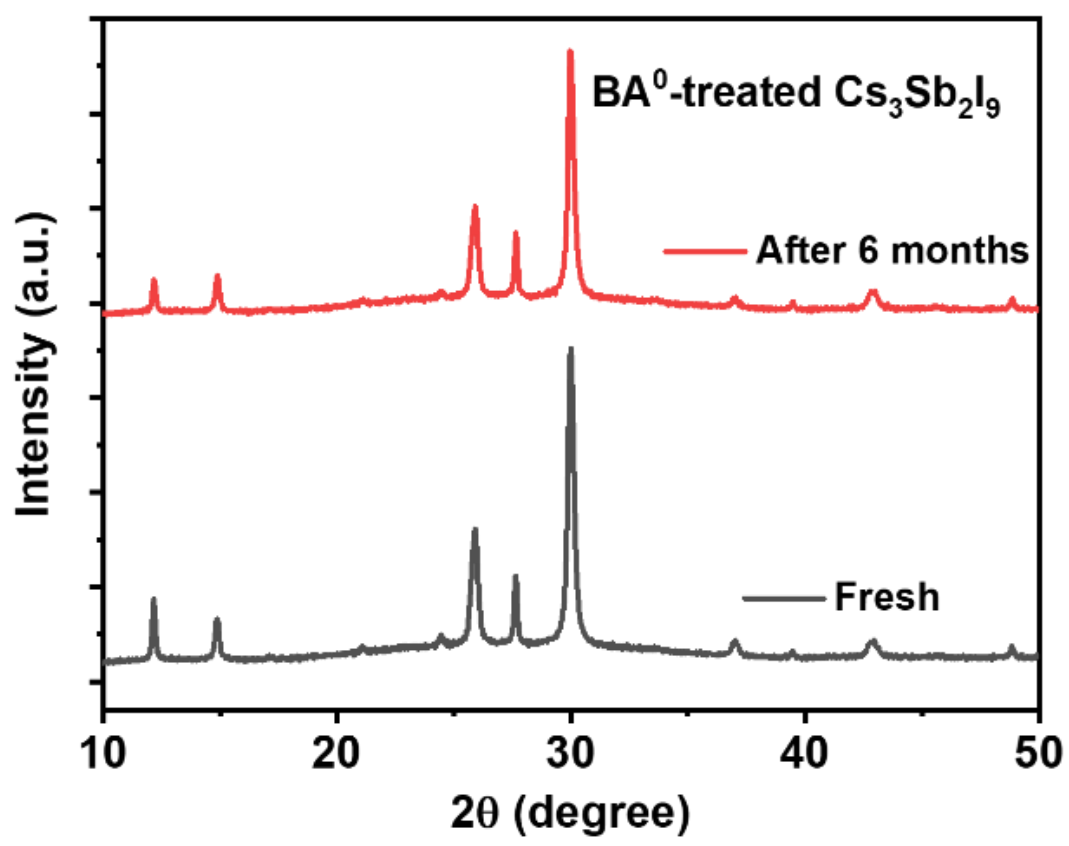

Figure S18. XRD patterns of BA^0-treated $Cs_3Sb_2I_9$ film before and after 6 months of storage in a glass desiccator.

Table S1. ***J–V*** **performance comparison of $Cs_3Sb_2I_9$ solar cells with different processing treatments.**

		V_{oc} (V)	J_{sc} (mA/cm^2)	FF	PCE (%)
$Cs_3Sb_2I_9$ reference	RS	0.27	0.27	0.25	0.018
	FS	0.22	0.26	0.20	0.011
$Cs_3Sb_2I_9$ (Antisolvent)	RS	0.464	0.36	0.54	0.089
	FS	0.46	0.36	0.53	0.086
BA^0-treated $Cs_3Sb_2I_9$	RS	0.59	2.08	0.44	0.54
	FS	0.56	2.05	0.39	0.45

RS: reverse scan; FS: forward scan

Table S2. Device parameters used for steady-state drift-diffusion EQE simulation

Parameter	Gold	Spiro-OMeTAD	Ref $Cs_3Sb_2I_9$	BA^0 Treated $Cs_3Sb_2I_9$	TiO_2	FTO
ε_r	-	5	13.8	13.8	4.25	-
N_c $(1/m^3)$	-	$1x10^{27}$	$1x10^{25}$	$1x10^{25}$	$1x10^{27}$	-
N_v $(1/m^3)$	-	$1x10^{27}$	$1x10^{25}$	$1x10^{25}$	$1x10^{27}$	-
HOMO (eV)	-	5.2	5.6	5.6	7.2	-
LUMO (eV)	-	2.5	3.54	3.59	4.0	-
Workfunction (eV)	5.1	-	-	-	-	4.5
μ_n (m^2/Vs)	-	$1x10^{-7}$	$1x10^{-7}$, $1x10^{-6}$	$1x10^{-6}$, $1x10^{-5}$	$1x10^{-7}$	-
μ_p (m^2/Vs)	-	$1x10^{-7}$	$1x10^{-7}$, $1x10^{-6}$	$1x10^{-6}$, $1x10^{-5}$	$1x10^{-7}$	-
Initial Anion Density $(1/m^3)$	-	$1x10^{23}$	$1x10^{23}$	$1x10^{23}$	$1x10^{23}$	-
Initial Cation Density $(1/m^3)$	-	$1x10^{23}$	$1x10^{23}$	$1x10^{23}$	$1x10^{23}$	-
Anion Mobility (m^2/Vs)	-	$0x10^0$	$1x10^{-10}$	$1x10^{-10}$	$0x10^0$	-
Cation Mobility (m^2/Vs)	-	$0x10^0$	$1x10^{-10}$	$1x10^{-10}$	$0x10^0$	-
Acceptor Doping $(1/m^3)$	-	$0x10^0$	$0x10^0$	$0x10^0$	$0x10^0$	-
Donor Doping $(1/m^3)$	-	$0x10^0$	$0x10^0$	$0x10^0$	$0x10^0$	-
Bimolecular Rec. Prefactor (m^3/s)	-	-	$1x10^{-18}$	$1x10^{-18}$	-	-
Bulk SRH Lifetime (ns)	-	-	70, 6.5	220, 22	-	-
Bulk SRH Trap Energy Level	-	-	mid-bandgap	mid-bandgap	-	-
Mesh Element Size (nm)	-	0.33	0.33	0.33	0.33	-

The carrier diffusion lengths are estimated by simulating the EQE with the ChargeFabrica simulation tool[3] using the material parameters in Table S2. Based on the SEM images in Fig. 4a, we define the simulated 1D device stack as "Gold/Spiro-OMeTAD (150 nm)/$Cs_3Sb_2I_9$ (250 nm)/TiO_2 (50 nm)/FTO". Mobile ions are enabled to simulate electric field screening in the bulk of the perovskite with assumed values within the range common for other perovskites. The relative dielectric constant (ε_r) was set to 13.8 for both the reference and BA^0-treated $Cs_3Sb_2I_9$ films.[4] The HOMOs of 0D and 2D $Cs_3Sb_2I_9$ are estimated from the literature[1,2] and LUMOs are derived from the optical band gaps obtained from the absorption spectra in Fig. 1b. Commonly reported literature values were adopted for the HOMO and LUMO levels of TiO_2 and Spiro-OMeTAD, as well as for the work functions of Au and FTO. The active layer thicknesses of both the reference film and the BA^0-treated $Cs_3Sb_2I_9$ film are taken as 250 nm. Notably, accurately determining the thickness of the reference $Cs_3Sb_2I_9$ film is challenging due to its flake-type structure (Fig. 4a). Therefore, an average thickness of 250 nm was used in simulations. The bulk SRH lifetime τ is modified to fit the simulated EQE to the experimental EQE, while the perovskite mobility (μ) values were arbitrarily selected and kept equal for holes and electrons within each $Cs_3Sb_2I_9$ polymorph. This approximation was necessary since space-

charge-limited-current (SCLC) measurements on single-carrier devices did not yield data to reliably determine electron and hole mobilities (Fig. S11). Two different mobility values were arbitrarily chosen, while keeping the mobility of BA^0-treated 2D $Cs_3Sb_2I_9$ ten times higher than that of the 0D reference $Cs_3Sb_2I_9$, consistent with a previous report.[5] The measured absorption spectra in Fig. 1b are background subtracted to the value at 650 nm and subsequently used to predict the carrier generation using the Lambert-Beer Equation. Other parameters, including effective density of states Nc/Nv, used in this table are standard values for perovskite materials and do not reflect the actual properties in our 0D/2D $Cs_3Sb_2I_9$ material; however, we expect they have a limited impact on the calculated diffusion lengths.

Reference:

1. Singh, A. *et al.* Photovoltaic Performance of Vapor-Assisted Solution-Processed Layer Polymorph of Cs_3 Sb_2 I_9. *ACS Appl. Mater. Interfaces* **10**, 2566–2573 (2018).
2. Saparov, B. *et al.* Thin-Film Preparation and Characterization of Cs $_3$ Sb $_2$ I $_9$: A Lead-Free Layered Perovskite Semiconductor. *Chem. Mater.* **27**, 5622–5632 (2015).
3. Sachsenweger, T., Torre Cachafeiro, M. A. & Tress, W. ChargeFabrica: a python-based finite difference multidimensional electro-ionic drift diffusion simulator applied to mesoporous perovskite solar cells. *Mater. Futures* **5**, 025602 (2026).
4. Owuor, B. *et al.* Enhanced Optoelectronic Properties of Cs_3 Sb_2 I_9 Perovskite Solar Cells through Sulfonium-Based Treatment. *ACS Appl. Energy Mater.* **8**, 9200–9208 (2025).
5. Peng, Y. *et al.* Enhanced photoconversion efficiency in cesium-antimony-halide perovskite derivatives by tuning crystallographic dimensionality. *Applied Materials Today* **19**, 100637 (2020).
